# Neurovascular coupling: a parallel implementation

**DOI:** 10.3389/fncom.2015.00109

**Published:** 2015-09-15

**Authors:** Katharina Dormanns, Richard G. Brown, Tim David

**Affiliations:** ^1^UC HPC Supercomputing Centre, University of CanterburyChristchurch, New Zealand; ^2^Institution of Mathematical Sciences, Massey UniversityPalmerston North, New Zealand

**Keywords:** neurovascular coupling, neurovascular unit, parallel computing, computational biology, agonistic behavior

## Abstract

A numerical model of neurovascular coupling (NVC) is presented based on neuronal activity coupled to vasodilation/contraction models via the astrocytic mediated perivascular K^+^ and the smooth muscle cell (SMC) Ca^2+^ pathway termed a neurovascular unit (NVU). Luminal agonists acting on P2Y receptors on the endothelial cell (EC) surface provide a flux of inositol trisphosphate (IP_3_) into the endothelial cytosol. This concentration of IP_3_ is transported via gap junctions between EC and SMC providing a source of sarcoplasmic derived Ca^2+^ in the SMC. The model is able to relate a neuronal input signal to the corresponding vessel reaction (contraction or dilation). A tissue slice consisting of blocks, each of which contain an NVU is connected to a space filling H-tree, simulating a perfusing arterial tree (vasculature) The model couples the NVUs to the vascular tree via a stretch mediated Ca^2+^ channel on both the EC and SMC. The SMC is induced to oscillate by increasing an agonist flux in the EC and hence increased IP_3_ induced Ca^2+^ from the SMC stores with the resulting calcium-induced calcium release (CICR) oscillation inhibiting NVC thereby relating blood flow to vessel contraction and dilation following neuronal activation. The coupling between the vasculature and the set of NVUs is relatively weak for the case with agonist induced where only the Ca^2+^ in cells inside the activated area becomes oscillatory however, the radii of vessels both inside and outside the activated area oscillate (albeit small for those outside). In addition the oscillation profile differs between coupled and decoupled states with the time required to refill the cytosol with decreasing Ca^2+^ and increasing frequency with coupling. The solution algorithm is shown to have excellent weak and strong scaling. Results have been generated for tissue slices containing up to 4096 blocks.

## 1. Introduction

The human brain is an immensely powerful organ that requires a delicately regulated supply of nutrients to sustain its activity. Unlike other organs in the body, cerebral tissue is unable to survive restricted blood supply for longer than a few seconds without cell death. The network of blood responsible for supplying the brain has two primary purposes, to deliver the required oxygen and nutrients to the tissue, and to convectively remove metabolic waste products such as CO_2_. Additionally, the blood vessel network has to maintain the blood supply under conditions of significant increases and decreases in blood pressure feeding the network such as occur when standing up from lying down, known as autoregulation, and additionally has to dynamically match supply to the varying metabolic demand of localized neuronal activity, a phenomenon known as functional hyperaemia or neurovascular coupling (NVC). Functional hyperaemia is an important mechanism which describes the local vessel dilation and constriction due to neuronal activity in the human/mammalian brain. The change in vessel diameter controls the local cerebral bloodflow and thereby the supply of oxygen and glucose. Impaired functional hyperaemia is associated with several pathologies such as hypertension, Alzheimer's Disease, cortical spreading depression, atherosclerosis and stroke (Iadecola, 2004; Girouard and Iadecola, 2006). All of these pathologies start with an altered relationship between neural activity and the cerebral blood flow (CBF). These alterations affect the delivery of substrates to active brain cells and impair the removal of waste-products from cerebral metabolism (Zlokovic, 2005). It is likely that this disruption contributes to brain dysfunction. Increasing the understanding of neural interactions highlights the importance of vascular pathology in cerebral diseases.

Although investigations of functional hyperaemia started over 100 years ago by Roy and Sherrington (1890), the exact cellular and chemical pathways that are involved are still unknown. However, studies over the last decades indicate that neurons, astrocytes, smooth muscle cells, and endothelial cells constitute a functional unit with the primary purpose of maintaining homeostasis in the cerebral micro-circulation (Iadecola, 2004; Hamel, 2006; Attwell et al., 2010; Drewes, 2012). Nutrient exchange primarily occurs in the capillaries, the fine mesh-like network of blood vessels embedded in the tissue, where species such as O_2_, CO_2_, and glucose are able to diffuse in and out of the capillary walls. The principal physical mechanism that controls bloodflow through the capillary network is the active dilation and contraction of the small arterioles that feed the tissue. Vessel dilation reduces the resistance to bloodflow, and hence increases the flow, while contraction restricts the blood supply. The contraction and dilation is due to the layers of smooth muscle cells surrounding the arteriole, which have the ability to actively contract or relax through actin-myosin crossbridge formation and cycling. The primary biochemical agent responsible for regulating this process is Ca^2+^ in the cytosol of the smooth muscle cell. Stimuli that have chemical pathways ultimately affecting cytosolic Ca^2+^ are manyfold, including shear stress at the arterial wall (Wiesner et al., 1997), transmural pressure in the vessel (Gonzalez-fernandez, 1994), tissue pH, and neuronal activity through glutamate and potassium release in the synaptic cleft (Attwell et al., 2010).

The cerebral cortex is fed with blood and nutrients from the outside inwards, starting at the pia mater (pial arteries) and bifurcating into the cortex with penetrating arteries which eventually perfuse the capillary bed. Thus, it is essentially a tree of blood vessels, which repeatedly bifurcates from a large root vessel, into vessels of shorter length and smaller radius. A cerebral vascular tree may comprise up to 20 or more bifurcation levels, corresponding to many millions of vessels in the tree. The dimensions of the vessels scale in such a way as to leave a significant fraction of the overall pressure drop from the root of the tree to the capillary bed across the smallest arterioles near the leaves of the tree (Fung, 1997). Variation in the resistance of these vessels can hence modify bloodflow in a highly localized manner, and collective variation can significantly modify the overall cerebral perfusion. Because of the connectivity of the tree, a change in resistance of one blood vessel can cause a change in pressure, and hence flow, throughout the binary tree. For example, if the resistance of blood vessels in a particular region decreases, then bloodflow will effectively be diverted through those vessels from other parts of the tree. The remainder of the tree will need, to some extent, to compensate for this decrease in flow. The effects that this connectedness will have on cerebral perfusion is not immediately apparent. One means of studying this problem is through large-scale simulation, whereby arteriole-level models of NVC (defined by a collection of linked cells as described below) incorporating the desired biochemical processes are inserted into a spatially-embedded vascular tree, and simulations are conducted at a macroscopic-scale.

These arterioles located within the cortex and providing nutrients of oxygen and glucose consist of the perfused lumen and a wall consisting of a thin layer of endothelial cells (EC) creating a tight boundary between the wall tissue and blood and covered by an outer layer of smooth muscle cells (SMC). The primary function of the SMC is to contract or relax, resulting in vessel constriction or dilation, respectively. The EC layer forms the blood brain barrier (BBB), preventing toxic, and metabolic waste products from diffusing into the cerebrospinal fluid (CSF). The assembly of cells which allow local perfusion of the cerebral tissue is collectively called a neurovascular unit (NVU), dynamically performing together and functioning as a major contributor to cerebral functional hyperaemia. For NVC signaling from the neuron to the SMC of the perfusing arteriole, glial cells are of particular importance, specifically astrocytes. These are known to have an important role in the control of the vessel diameter.

The astrocyte is a star-shaped cell with peripheral ends located near the synaptic clefts of neurons and other astrocytes whilst their end-feet of the astrocyte are located in a layered fashion around vascular smooth muscle cells (VSMC). Here the gap between astrocytic endfeet and VSMCs is known as the perivascular space. One important function of the astrocyte is their ability to buffer extracellular K^+^ and other neurotransmitters. These buffered ions can be taken up and transported to the end-feet of the astrocyte and released into the perivascular space and subsequently taken up by the SMC of the perfusing arteriole.

**Figure 1:**
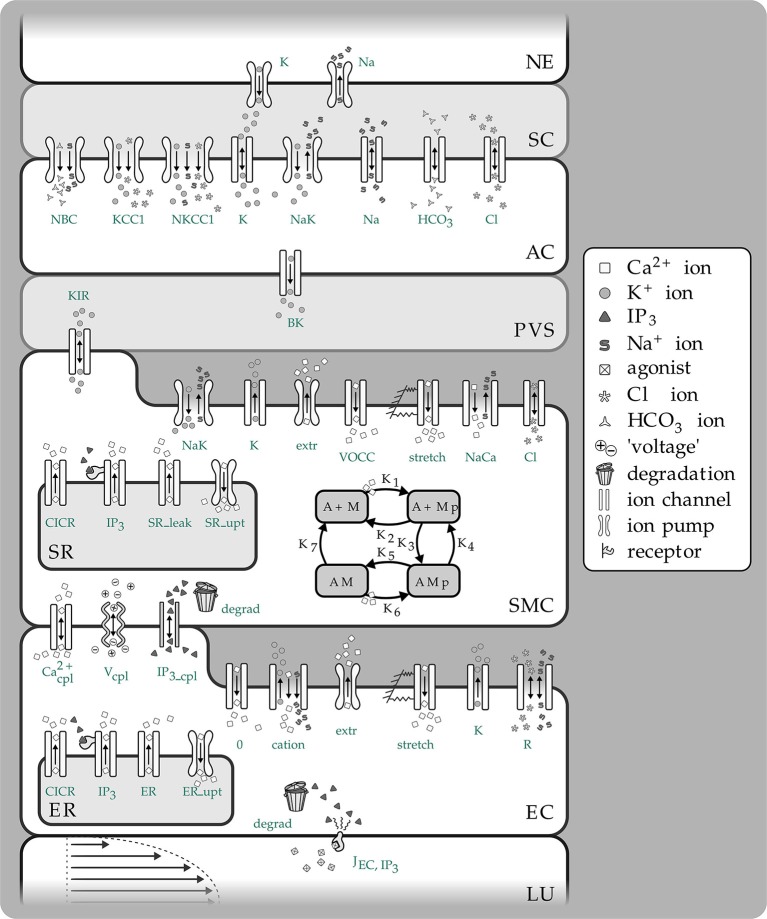
**Overview of the complete NVC Model including all subsystems**. NE, Neuron; SC, Synaptic cleft; AC, Astrocyte; PVS, Perivascular space; SMC, Smooth muscle cell; SR, Sarcoplasmatic reticulum; EC, Endothelial cell; ER, Endoplasmatic reticulum; LU, Lumen; NBC, Sodium bicarbonate pump; KCC1, Potassium chloride cotransporter pump; NKCC1, Sodium potassium chloride cotransporter pump; BK, Large conductance potassium channel; VOCC, Voltage-operated calcium channel; CICR, Calcium induced calcium release channel; R, Residual current regrouping channel; K_1_, K_7_, reaction rate constants; M, free nonphosphorylated cross bridges; Mp, free phosphorylated cross bridges; AMp, attached phosphorylated cross bridges; AM, attached dephosphorylated latch bridges.

The endothelial and SMC have intracellular communication via hetero-cellular connexin gap-junctions (Haddock et al., 2006). Cells can change their dynamical state by transporting messenger molecules such as IP_3_ through these gap-junctions. Indeed as noted below IP_3_ is a mediator in the calcium-induced-calcium-release (CICR) mechanism which can induce oscillations in the SMC. Additionally EC are able to detect and respond to changes in the radial change through stretch-activated channels. This is shown in Figure 1 in the EC and denoted as “stretch.” The channel provides a flux of Ca^2+^ into the EC which through a gap junction transfers Ca^2+^ to the SMC causing constriction via the myocin-actin pathway. The stretch channel flux equation is a function of a number of variables, notably the membrane potential and the vessel radius and is given as

(1.1) $$J_{stretch_{i}}=\frac{G_{stretch}}{1+exp\left(-\alpha_{stretch}\left(\frac{\Delta pR}{h}-\sigma_{0}\right)\right)}\left(v_{i}-E_{SAC}\right)\;.$$

Here *p* is the pressure in the vessel, *R* the radius *G*_stretch_ the channel conductance, h the vessel wall thickness, *E*_*SAC*_ the Nernst (equilibrium) potential, α_stretch_ a model parameter and σ_0_ a circumferential stress associated with an equilibrium state of the vascular tone of the vessel. Hence this is essentially the link between the NVU and vascular tree. The center of the NVU is the SMC since as noted above it receives input from both neuronal activity via the astrocyte and indications of the state of bloodflow from the endothelium. These inputs are in the form of voltage coupling via ionic transport, release of SMC cytosolic Ca^2+^ from the sarcoplasmic reticulum due to IP_3_ transported from the EC, and finally Ca^2+^ from the EC itself. Investigating the relationship between neuronal activity and the resulting variation in the radius of the perfusing arteriole in addition to the role of EC/SMC connectivity and the resulting effects of flowing blood is crucial to the further understanding of NVC and how it interacts over the larger scale elucidated by non-invasive measurement techniques such as fMRI; our current multi-scale model provides a framework for investigating both scales simultaneously. The complex NVU model used is that described in Dormanns et al. (2015). The model is based on experimentally validated ion channel parameters providing the model with the ability to describe the full NVC phenomenon. The model contains that which we believe to be the fundamental ingredients of NVC but it also has the ability to include other pathways.

The perfusion of blood provides not only oxygen and glucose (a natural nutrient) but also through the vasculature a spatially varying concentration of agonists via convection and the release of ATP from red blood cells by fluid shear stress. P2Y receptors situated on the luminal side of the EC and activated by these agonists such as ATP provide an IP_3_ signaling pathway via the membrane bound phospholipase-C (PLC) (Keizer and De Young, 1992) thus allowing information on the bloodflow environment to pass to the outer SMC layers through the heterotypic gap-junctions.

Up to now *in vitro, in vivo*, and *in silico* experiments have either investigated time-dependent flow through either simple or complex vasculatures or looked at cell functioning *independent* of the consideration of how blood flow itself is affecting the neural phenomena and vice versa.

To investigate the effect of complex cellular models embedded in a vascular tree we allow a single NVU to be connected to each of the leaves of a tree which models in some topological sense a small section of cortical tissue/vasculature. Thus, an individual NVU perfuses a small section of tissue. The activity of each NVU may be varied according to its position in the tissue block. By introducing a model of a spatially embedded dynamic vascular tree regulated by a time-dependent and spatially varying cellular scale model of NVC we can begin to examine some of the effects introduced by the connectivity of the structure of the tree and the dynamics of the cellular function. In addition the coupling induced by the vascular tree presents numerical challenges to not only the numerical solution of the resulting ODE or PDE systems but also because of the possible size of the system to the parallel implementation itself. Dormanns et al. (2015) showed that for certain concentrations of agonist in both the flowing blood and tissue the EC/SMC system exhibits domains of oscillation and induces oscillation (or vasomotion) in the arteriolar radii of the vascular tree. In addition the frequency changes as a function of agonist concentration. The model is therefore able to investigate the relationship between oscillatory states of the EC/SMC and the resulting motion of the vasculature.

## 2. Materials and methods

In this section we first describe the basic structure of the vascular tree which allows for a space filling simulation. The root of the tree can be considered as either a pial artery and a subsequent penetrating arterial structure into the cerebral cortex or the start of the penetrating structure itself. Having determined the basis of the tree the NVU is briefly described but as noted the details can be found in Dormanns et al. (2015). Finally a description is given which links the two models together forming a multi-scale model and an algorithm which determines the numerical solution method in a parallel environment.

### 2.1. Vascular tree model

The vascular network considered in this article is a tree-like structure of blood vessels modeling a portion of the cerebral vasculature branched off from the large cerebral arteries. The blood vessels branch into successively shorter and narrower vessels until they reach the fine mesh structure of the capillary bed. The final vessels in the tree are referred to as the terminal arterioles, and have a typical radii of 10 μ m. The Reynolds and Womersley numbers for the flow in the small blood vessels in the model are very small, so we can readily assume a Hagen- Poiseuille flow: for a vessel of length *L* and radius *R*, we have

(2.1) $$Q=\frac{\pi R^{4}}{8\mu L}\Delta P\;,$$

where *Q* is the blood flow, μ is the blood viscosity and Δ*P* is the pressure drop over the vessel. We fix characteristic length and pressure values *R*_0_, *L*_0_, and *P*_0_, and setting *R* = *R*_0_*r*, Δ*P* = *P*_0_*w*, and *L* = *L*_0_*l*, Equation (2.1) becomes

(2.2) $$Q=\frac{\pi R_{0}^{4}P_{0}}{8\mu L_{0}}\frac{r^{4}}{l}w=Q_{0}gw=Q_{0}q\;,$$

where *g* = *r*^4^∕*l* is the (non-dimensional) conductance of the vessel. The equation *q* = *gw* is analogous to Ohm's Law for an electrical circuit, with flow *q* taking the role of current, *w* as potential difference, and *g* as conductance (reciprocal of resistance). Note that the conductance is highly sensitive to the radius of the vessel. Because most of the systemic pressure drop occurs over the small arterioles, we assume a constant capillary bed pressure *p*_cap_, and a (possibly) time-dependent pressure *p*_0_(*t*) at the root of the tree.

#### 2.1.1. Spatially embedded vascular tree

As a crude approximation of a tree with physiologically realistic morphology, we model the tree as a symmetric binary tree, with a 2D H-tree spatial structure. At each bifurcation, the radius of the daughter vessels is a factor α of the radius of the parent, and the length is divided by 2 every second bifurcation. These scalings are again within the biologically realistic limits described in the asymetric binary tree algorithm of Ottesen et al. (2004). The smallest vessels are defined to have radius 10 and length 200 μ m, and the scale of the model is therefore determined by the number of levels in the tree. Finally, we assume that when unpressurized, each vessel has wall thickness h^*^ proportional to its radius R^*^, namely

(2.3) $$h^{*}=h_{RR}R^{*}\;.$$

The result is a tree with a somewhat realistic morphology, but with a space-filling property that enables the coupling of 2D or 3D spatial phenomena such as the localized neural activity to the inherently 1D (or 0D) problem of blood flow in the vascular tree.

### 2.2. Neurovascular coupling model

The model used to describe the NVC is that of Farr and David (2011) and Dormanns et al. (2015). We do not describe the full details but refer the reader to Dormanns et al. (2015) however in the interests of continuity a short description of the model and its subsystems is given below. The full NVU model is divided into seven basic compartments: the neuron (NE), the synaptic cleft (SC), the astrocyte (AC), the perivascular space (PVS), the smooth muscle (SMC), and endothelial (EC) compartments and the arteriolar lumen (LU). These compartments are shown in Figure 1. These compartments along with the synaptic cleft and the perivascular space are represented in the model by separate subdomains. These subdomains are assembled together using a lumped parameter approach where spatial variations in the compartment are considered negligible, thus allowing intercellular interactions. We should note that although each compartment is assumed to contain a number of homotypic cells and to have differing volumes they are considered to be an aggregate of cells and therefore act as a single entity. Each compartment contains a number of ion channels allowing fluxes of ions, such as Ca^2+^ as well as second messenger molecules (e.g., IP_3_). This allows for the formation of conservation equations for ions, other molecular species and through the use of Kirchoff's Law the evaluation of the membrane potential for each cell type. Heterotypic gap junctions are also modeled between the smooth muscle and EC thus simulating the connectivity of the arterial wall. A graphical overview of these compartments and the channels is shown in Figure 1.

To understand the connectivity of the full system we consider four “subsystems”, the Neuron/Astrocyte subsystem (NE/AC) including the PVS and SC, the Smooth Muscle Cell and Endothelial Cell subsystem (SMC/EC) which couples the SMC and EC together, the Arteriolar Contraction subsystem and the Arteriolar Wall Mechanical subsystem. We treat each subsystem as having a “triggering” input and subsequent output that provides connectivity and a further “triggering” input to the other linked subsystems, essentially a coupling influence across all sub-systems providing a holistic model.

### 2.3. NE/AC subsystem

The model for this subsystem extends the model of Østby et al. (2009) by adding a large conductance potassium channel in the astrocytic wall and directing K^+^ into the perivascular space with flux *J*_*BK*_. The Østby model was chosen since it provides the basic model for potassium efflux. We use this on the basis of Filosa's work which showed that K^+^ efflux into the synaptic cleft is one of the dominant effects of neuronal activity. Farr and David (2011) assumed that in addition to K^+^ glutamate is produced (Attwell et al., 2010). For brevity we do not show details of Østby's complete model but simply refer the reader to Østby et al. (2009) and also to the Supplementary Material, which contains a full list of equations used. We have used the model of Østby for neuronal activity rather and although simple provides the required time-dependent input for the NE/AC subsystem, treated as a pulse like release of K^+^ into the synaptic cleft and a simultaneous equal flux back into the neuron via the NaK-ATPase pump. The total input signal, *f*(*t*), and corresponding increased K^+^ concentration in the synaptic cleft are shown in Figure 2 in Dormanns et al. (2015).

Beside this neuronal input signal the NKCC1 (sodium-potassium-chloride) and KCC1 (potassium-chloride) co-transporters are only enabled when the neuronal ion release and spatial buffering are applied. This behavior is modeled by a simple step function with the value 1 when both are activated and with a default value of 0. This co-transporter behavior is shown by the red dashed line in Figure 2 in Dormanns et al. (2015).

The increase of K^+^ in the synaptic cleft results in an increased K^+^ uptake by the astrocyte, which consequently undergoes depolarization. This results in a K^+^ efflux in order to repolarize the compartment (“cell”) membrane back to its steady state potential. Since physiologically most of the astrocytes' K^+^ conductance channels are located at the end-feet, the outward current-carrying K^+^ would flow out largely through these processes. These end-feet K^+^ conductance channels are modeled by the BK channel flux (see Dormanns et al., 2015 for details). Consequently, the K^+^ is “siphoned” from the synaptic cleft to the end-feet of the astrocyte and released into the perivascular space by the BK channel. This efflux increases the perivascular space K^+^ concentration which is an input variable for the SMC/EC subsystem.

### 2.4. SMC/EC subsystem

The SMC/EC subsystem model extends the work of Koenigsberger et al. (2006) by adding an inward-rectifying potassium (KIR) channel at the interface between the SMC and the PVS. There are effectively two inputs to this subsystem, the KIR channel on the SMC facing the PVS and allowing a flux of K^+^ into the cytosol and the influx of IP_3_ into the EC by virtue of the luminal agonist P2Y receptors on the EC membrane. This receptor is activated by a number of agonists flowing in the blood, for example ATP derived from the red blood cells under shear stress.

The KIR channel is mediated by K^+^ concentration in the perivascular space [K^+^]_*p*_, which varies after neuronal activity. The rise in K^+^ activates the KIR channel on the SMC causing extrusion of more potassium into the PVS. The efflux of cytosolic K^+^ via the KIR channel hyperpolarizes the smooth muscle, causing the voltage-operated Ca^2+^ channel to close and preventing any further influx of Ca^2+^ into the smooth muscle cytosol. The formulation of the KIR channel uses the data of Filosa et al. (2006).

The second input to the SMC/EC subsystem is that of IP_3_ generation in the EC due to the activation of membrane receptors by agonists flowing in the arteriolar lumen. IP_3_ mediates the channel in both the EC and SMC, situated on the surface of the endoplasmic (EC) and sarcoplasmic reticulum (SMC). This allows Ca^2+^ to be released from the reticula. With certain IP_3_ concentrations inside either the SMC or EC compartments, Ca^2+^ oscillations can occur due to the calcium induced calcium release (CICR) process of Goldbeter et al. (1990). The production of IP_3_ in the EC is a function of the activity of the P2Y receptors which is mediated by the concentration of an agonist (in this case ATP) at the cerebral vascular lumen and in the case of this model also in the tissue. We treat the ATP concentration as constant within the lumen compartment and tissue but allowing it to be spatially varying within the tissue block perfused by the vascular H-tree defined above.

Physiologically, ECs and SMCs are connected by hetero- and homo-cellular gap junctions that allow an intercellular exchange of molecules and voltage. The heterocellular exchange is implemented by linearized coupling fluxes such that the flux is simply a function of the difference of concentrations (or membrane potential) between EC and SMC. These coupling functions are given by

(2.4) $$\begin{array}{rcl} J_{Ca_{cpl}^{2+}} & = & -P_{Ca^{2+}}\left({\left[Ca^{2+}\right]}_{i}-{\left[Ca^{2+}\right]}_{j}\right)\\ J_{V_{cpl}} & = & -G_{v}\left(V_{i}-V_{j}\right)\\ J_{IP_{3_{cpl}}} & = & -P_{IP_{3}}\left({\left[IP_{3}\right]}_{i}-{\left[IP_{3}\right]}_{j}\right)\text{,} \end{array}$$

where the subscripts i,j correspond EC and SMC, respectively. Barrio et al. (1997) investigated the voltage-gating properties of connexin-43 junctions and indicated that the conductance of the hemi-channel was voltage mediated. However, for this particular model, we treat $P_{Ca^{2+}},G_{v}$, and $P_{IP_{3}}$ as constants, this can be seen as modeling the simple diffusional flux rather than the more complete electro-diffusional flux which allows for ion drift. We choose, for the results presented here, the set of coupling coefficients for Case 2 found in Dormanns et al. (2015). It should be noted that this coupling only exists internally to each tissue block. For the cases and results presented here no coupling exists between the tissue to simulate effective diffusion (this will be the subject of a future paper). The perfusing artery, as a compartment of the NVC model, is treated as a single time-dependent variable, that of the radius of the artery. This radial change provides the link to the NVU with the vascular tree.

### 2.5. The arteriolar contraction subsystem

The formation of cross bridges between the actin and myosin filaments in a SMC provides the contraction force and is mediated by cytosolic Ca^2+^. The arteriolar contraction subsystem model is based on the work of Hai and Murphy (1989), and uses the SMC compartmental cytosolic Ca^2+^ concentration as input signal.

There are four possible states for the formation of myosin: free nonphosphorylated cross bridges (M), free phosphorylated cross bridges (Mp), attached phosphorylated cross bridges (AMp) and attached dephosphorylated latch bridges (AM). The dynamics of the fraction of myosin in a particular state is given by four differential equations:

(2.5) $$\begin{array}{l} \frac{\text{d}[M]}{\text{d}t}=-K_{1}[M]+K_{2}[Mp]+K_{7}[AM]\\ \frac{\text{d}[Mp]}{\text{d}t}=K_{4}[AMp]+K_{1}[M]-(K_{2}+K_{3})[Mp]\\ \frac{\text{d}[AMp]}{\text{d}t}=K_{3}[Mp]+K_{6}[AM]-(K_{4}+K_{5})[AMp]\\ \frac{\text{d}[AM]}{\text{d}t}=K_{5}[AMp]-(K_{7}+K_{6})[AM] \end{array}$$

with

(2.6) [AM]+[AMp]+[Mp]+[M]=1 ,

where the rate constants, *K*_*n*_ (*n* = 1, …, 7), regulate the phosphorylation and bridge formation. With Equation (2.6) we need only solve for [*AM*], [*AMp*], and [*Mp*].

The Ca^2+^ dependence of the cross bridge model is modeled by rate constants *K*_1_ and *K*_6_. The total phosphorylation of myosin is a function of the SMC compartmental Ca^2+^ (Koenigsberger et al., 2006) so that *K*_1_ and *K*_6_ are given by:

(2.7) $$K_{1}=K_{6}=\gamma_{\text{cross}}{\left[Ca^{2+}\right]}_{i}^{3}$$

in which γ_*cross*_ is a constant characterizing the Ca^2+^ sensitivity of calcium-activated phosphorylation of myosin.

The active stress of a SMC is directly proportional to *F*_*r*_, the fraction of attached cross bridges, and is given by Equation (2.8). This is used as input parameter for the Mechanical subsystem.

(2.8) $$F_{r}=\frac{\left[AMp]+[AM\right]}{{\left(\left[AMp]+[AM\right]\right)}_{\text{max}}}$$

### 2.6. The arteriolar wall mechanical subsystem

As an initial and simple representation the basis for the Mechanical subsystem is a Kelvin-Voigt model, which describes the visco-elastic mechanical behavior of the arterial wall. The model consists of a Newtonian damper and Hookean elastic spring connected in parallel. The fraction of attached myosin cross bridges as described in Equation (2.8), *F*_*r*_, is the input signal for the Mechanical subsystem and corresponds with the active stress state of the SMC in the circumferential direction.

This circumferential stress in the arterial wall, σ_θθ_, is given by:

(2.9) $$\sigma_{\theta \theta }=E\epsilon_{\theta \theta }+\eta \frac{\text{d}\epsilon_{\theta \theta }}{\text{d}t}\text{ ,}$$

where *E* is the Young's modulus, η the viscosity and ϵ_θθ_ the strain in the arterial wall. Assuming that the acceleration of the vessel wall due to changes in σ_θθ_ is negligible, Laplace's law is used in order to relate the circumferential stress to the change in radius:

(2.10) $$\sigma_{\theta \theta }=\frac{R\Delta p}{h}\text{ ,}$$

where Δ*p* is the transmural pressure, *R* the vessel radius and *h* the vessel thickness. For simplicity we treat the wall thickness as a constant fraction of the radius, *h* = 0.1*R*.

To obtain the Young's modulus, *E*, and initial radius, *R*_0_, as a function of the attached myosin cross-bridges, experimental data of Gore and Davis (1984) is used. For the Mechanical subsystem, a linear function is utilized mapping the fully activated state to the fully relaxed state. The linear fit is based on the radii between 10 and 30 μ m, since the full model only models small strains.

The Young's modulus and initial radius is assumed to be a continuous function of *F*_*r*_ and a linear interpolation is used between two known experimental data states (active *E*_*act*_ and passive *E*_*pas*_) taken from Gore and Davis (1984) of the SMC. We can therefore write

(2.11) $$E(F_{r})=E_{pas}+F_{r}\left(E_{act}-E_{pas}\right)\;.$$

In a similar manner the initial radius is given by

(2.12) $$R_{0}(F_{r})=R_{0_{pas}}+F_{r}\left(R_{0_{act}}-R_{0_{pas}}\right)\;.$$

From Equation (2.9), using Laplacian's law (Equation 2.10), and Equations (2.11, 2.12) for the Young's modulus and initial radius, respectively, as function of the *F*_*r*_, an expression for the time-dependent vessel radius can be derived:

(2.13) $$\frac{R\Delta p}{h}=E(F_{r})\left(\frac{R-R_{0}(F_{r})}{R_{0}(F_{r})}\right)+\eta \frac{\text{d}}{\text{d}t}\left(\frac{R-R_{0}(F_{r})}{R_{0}(F_{r})}\right)\;,$$

giving

(2.14) $$\frac{\text{d}R}{\text{d}t}=\frac{R_{0_{pas}}}{\eta }\left(\frac{R\Delta p}{h}-E(F_{r})\frac{R-R_{0}(F_{r})}{R_{0}(F_{r})}\right)\;.$$

### 2.7. Numerical solution method

A total of 24 coupled ordinary differential equations (ODEs) make up the present NVU system and are solved using a C implementation of a backward Euler integration with Newton iteration due to the domains of stiffness encountered within the different cases outlined in the Section Results.

### 2.8. Parallel implementation

The parallel implementation follows the work of Brown (2013), however we give a brief explanation here for brevity. Because of the global coupling induced by the resistive network, the resulting stiff system of ODEs has a dense Jacobian which precludes the direct application of traditional implicit methods for numerically solving the differential equations. We consider a technique for remedying this problem by taking a block diagonal Jacobian approximation which allows use of an implicit method but retains the desirable property of explicit solvers of linear solution time scaling with problem size. Additionally, the method is amenable to significant parallelization, and we present scaling results. A mathematical description of the problem is as follows. We model the network by a directed graph with *m* internal nodes and *n* edges. The graph is represented by an incidence matrix *A* ∈ ℝ^*m*×*n*^ with entries *a*_*ij*_ given by: *a*_*ij*_ = −1 if edge *j* enters node *i*, *a*_*ij*_ = 1 if edge *j* exits node *i*, and *a*_*ij*_ = 0 otherwise. With imposed potential or flow boundary conditions, the potential differences ***w*** across the edges are given by an expression of the form

(2.15) $$w=A^{T}p+b\;,$$

where ***p*** ∈ ℝ^*m*^ is the vector of potentials at each internal node, and ***b*** ∈ ℝ^*n*^ incorporates the boundary conditions.

We assume that the constitutive relation on each edge is Ohm's law: the flow through edge *j* is *q*_*j*_ = *g*_*j*_*w*_*j*_ where *g*_*j*_ is the conductance of the edge. In matrix form,

(2.16) $$q=Gw\in {\mathbb{R}}^{n}\;,$$

where *G* = diag *g*.

Flow conservation requires *A*q=0, and thus the vector of potentials ***p*** ∈ ℝ^*m*^ is given by solving the positive definite linear system

(2.17) $$AGA^{T}p=-AGb\;.$$

The class of models we consider takes such a network and couples it to a system of ordinary differential equations (ODEs)

(2.18) $$\dot{x}=f(x,p,t),\;\;\;\;\;x\in {\mathbb{R}}^{\text{l}},\text{l}\in \mathbb{N}\;,$$

where the vector ***x*** corresponds the set of state variables of the ODE system. The differential equations for the state variables depend on the potentials in the network, and the conductances in the network are algebraically dependent on the state variables, *g*=*g*(***x***) (the conductances can be computed from the radii of the terminal arterioles). Further, the boundary conditions may be time-varying, ***b***=***b***(*t*). Equations (2.17, 2.18) thus define a semi-explicit index-1 differential algebraic system of equations. The system can naively be solved by a standard ODE methods by transforming it into an ODE system:

(2.19) $$\frac{dx}{dt}=f(x,\;{\left(AG(x)A^{T}\right)}^{-1}AG(x)b(t),\;t)$$

The linear system (Equation 2.17) is positive definite, typically highly sparse, and depending on its structure, can be efficiently solved by the conjugate gradient method or direct sparse Cholesky factorization. Hence, function evaluations for this ODE system are relatively cheap. However, the system is stiff, requiring the evaluation of the Jacobian of the system. The Jacobian of Equation (2.19) can be decomposed into the following form:

(2.20) $$J=\frac{\partial f}{\partial x}+\frac{\partial f}{\partial p}\frac{\partial p}{\partial g}\frac{\partial g}{\partial x}\;.$$

For a typical problem the terms $\frac{\partial f}{\partial x}$,$\frac{\partial f}{\partial p}$, $\frac{\partial g}{\partial x}$ are likely to be highly sparse, because *f* and *x* span the all of the tissue blocks and components, e.g., $\frac{\partial f_{i}}{\partial x_{j}}$ in these matrices are only non-zero if *i, j* correspond to equations / variables within the same tissue block. The term $\frac{\partial p}{\partial g}$ is given by

(2.21) $$\frac{\partial p}{\partial g}=-(A\text{ diag}{\left(g(x)A^{T}\right)}^{-1}A\text{ diag}(A^{T}p+b)\;,$$

which is a dense matrix, since it contains the inverse of a sparse matrix *A* diag(***g***)*A*^*T*^ and that matrix cannot be permuted into block triangular form. The system Jacobian *J* is thus also dense, precluding its direct evaluation for a large-scale problem. For large-scale problems it is not feasible to construct the full Jacobian of the system to use in a direct linear solver. For example, the autoregulation problem for a vasculature involving approximately 10,000,000 vessels would require more than 20 TB of RAM to store the Jacobian. We choose the approximation to have block diagonal structure, the factorization and solution can be decomposed into as many independent tasks as there are blocks, and hence the decomposition yields a natural parallelization of the problem. Details of this procedure can be found in Brown (2013).

#### 2.8.1. Parallel considerations

When this algorithm is implemented on a parallel system, the H-tree network is partitioned at a coarser scale, one subnetwork per computational node. Each of these coarse partitions is formed from the union of a number of fine partitions. Each coarse partition has its own incidence matrix *A*^(*i*)^ with its own set of boundary values ***b***^(*i*)^ corresponding to where it meets the problem boundary and other subnetworks. Before the approximate Jacobian can be evaluated, a global computation of pressures throughout the network is completed.

#### 2.8.2. Large-scale simulation of the neurovascular coupling model

We now consider applying the methodology to our previously described autoregulation model. The tissue blocks and terminal arterioles are of fixed size, so the overall problem size is dictated by the number of levels, *L*, in the binary tree.

**Figure 2:**
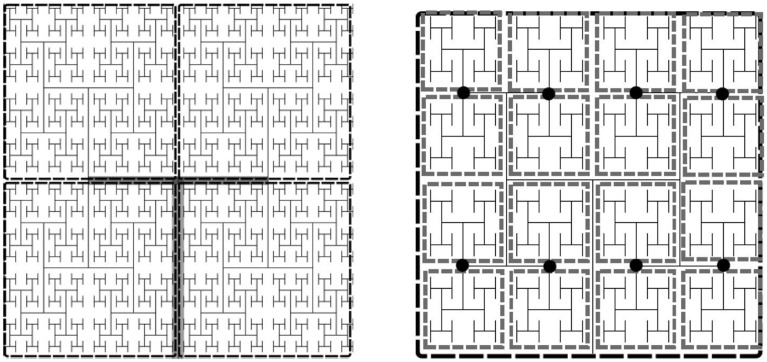
**Example of an H-tree model of the vasculature showing fine and coarse decomposition**. **Left:** Depicts an approximately 12 × 12 × 0.4 mm tissue slice. Each subdomain of the coarse partition is mapped to one processor, whereas the fine partitioning of each subdomain sets the Jacobian block size. For this example *N*_*S*_ = 5, so there are 16 tissue blocks and hence 16 × 24 state variables per fine partition, the Jacobian block size is 384 × 384. **Left:** Tree of *L* levels has an m × n incidence with *L* = 11 into *N* = 4 four subtrees (*L*_0_ = 2). The root subtree is shown in gray. **Right:** Fine partitioning of subtree with *N*_*S*_ = 5 for Jacobian approximation. The network is split at the nodes (marked as black circles), resulting in Jacobian blocks for the state variables in each gray rectangle.

To parallelize the problem, the tissue domain is split into *N* subdomains, where $N=2^{L_{0}},L_{0}\in \mathbb{N}$, where *L*_0_ is the number of levels in the root subtree such that each of the subdomains corresponds to an equally sized subtree with incidence matrix *A*^(*i*)^. The root subtree of the network connecting each of these subtrees, has incidence matrix *A*^(0)^. The subtrees are binary trees of *L*−*L*_0_ levels each. For each subtree in the coarse partition we specify the fine partitioning for the Jacobian approximation by further decomposing each subtree into its own root tree with subtrees of *N*_*S*_ levels. The imposed boundary nodes are the nodes where the fine subtrees interface with the root. The degree of sparsity of the Jacobian can be controlled by specifying the number of levels in the tree, *N*_*S*_, each subtree spans. The two decompositions are illustrated in Figure 2.

### 2.9. Implementation

Numerical experiments were conducted to assess the convergence and scalability of the approach for the cerebral vasculature problem in a moderate sized SMP cluster. The test system used is 8 nodes (up to 256 processors) of the BlueFern IBM Power 755 cluster at the University of Canterbury. Intranodal communication is conducted using shared memory, while internodal communication utilizes an Infiniband network. To solve the ODE system, a fixed-step Backward Euler method was used to solve the radial change of the leaf of the H-tree using the NVC model described above in Section 2.2. The Jacobian was computed from the initial state, and then updated subsequent to any iteration that took more than 8 iterations to converge. Newton's method was said to converge when either the function value or relative error dropped below a tolerance of ϵ = 10^−6^. The matrices ∂***f***^(*i*)^∕∂***p***^(*i*)^ and ∂***f***^(*i*)^∕∂*x*^(*i*)^ were computed numerically by finite differences, using the algorithm of Curtis et al. (1974). The equations were all scaled to have steady state values near 1. The matrix ∂***g***^(*i*)^∕∂***p***^(*i*)^ was computed analytically. The algorithm was implemented in C with MPI, compiled using IBM's XLC compiler, and utilized CSparse (Davis, 2006) for the sparse matrix operations (sparse Cholesky factorization, sparse LU decomposition, and matrix multiplication). The code was executed on a number of cores ranging from 8 to 256.

## 3. Results

### 3.1. Numerical scaling experiments

**Figure 3:**
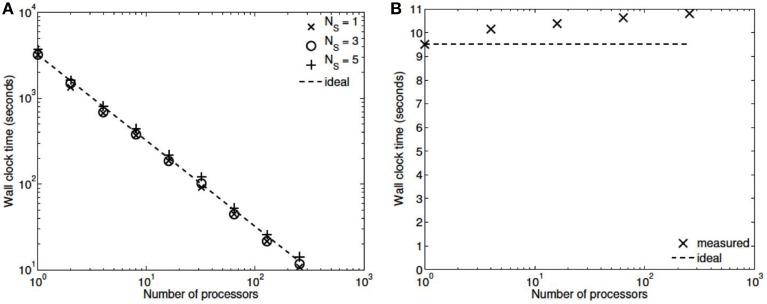
**(A)** Strong scaling (*L* = 23, *N*_*S*_ = 1,3,5; *N* = 1,2,4,8,…,256). **(B)** Weak scaling (*L* = 15,…,23; *N* = 1,…,256, *N*_*S*_ = 3).

**Figure 4:**
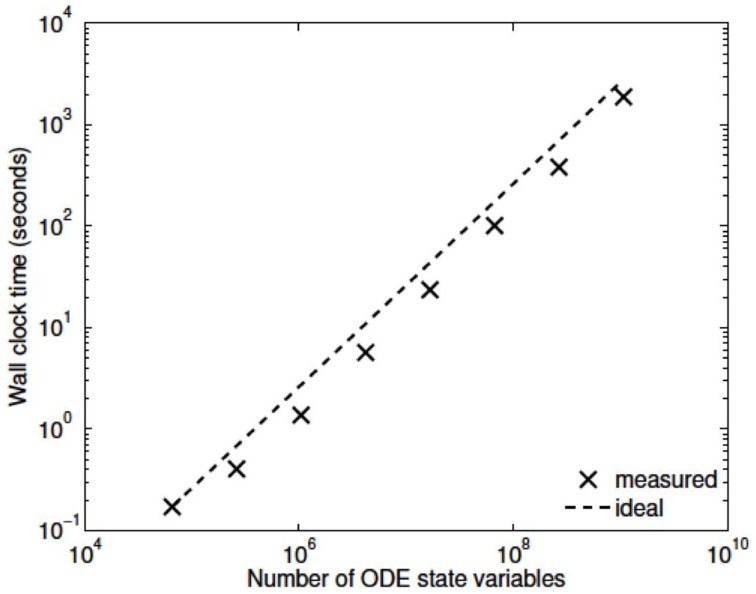
**Scaling with problem size (***L*** = 15,…,29, ***N*** = 128, ***N***_***S***_ = 3)**.

Three numerical experiments were performed in order to determine the scaling effects of the parallel code, the results of which are depicted in Figure 3. The first experiment measured the strong scaling performance of the problem for a fixed problem size of *L* = 23 levels, with subtree sizes *N*_*S*_ ∈ 1, 3, 5. The number of processors was varied in powers of 2 between 1 and 256 and the simulation wall clock time recorded. Figure 3A shows that up to this number of processors, the method scales ideally. Scaling is initially superlinear, because initially as more processors are added, more of the problem is able to fit in fast cache memory. Increasing the subtree size increases the computational time, but does not affect the scalability, which is dominated by the communication. The second experiment measured the weak scaling performance of the problem, whereby the problem size is increased in proportion with the number of processors. The problem size was varied between *L* = 15 and *L* = 23, with the number of processors varying between *N* = 1 and *N* = 256. Figure 3B shows that the elapsed computational time increases slightly as the problem and computing size increases. Small problem sizes were chosen to accentuate the relative contribution of communication, increasing the problem scale would flatten the curve further. Finally, the performance of the method was tested with a fixed number of processors (*N* = 128) and problem size varying between *L* = 15 and *L* = 29. The imposed block-diagonal structure would be expected to provide linear solution time with respect to problem size because the Jacobian evaluation and factorization is linear in the number of blocks, as is the evaluation of a Newton step. Because the number of processors is relatively small, the amount of time spent in communication is very small relative to the computation. The measured scaling results are depicted in Figure 4, and reflect the expected linear performance.

### 3.2. Multi-scale simulations

**Figure 5:**
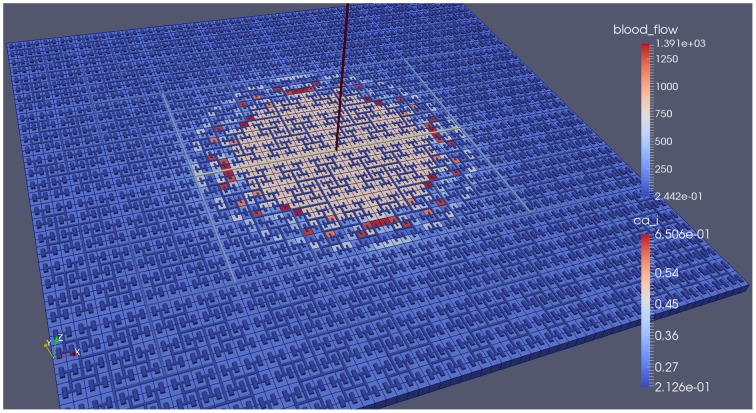
**Simulation of a 25.6 × 25.6 mm cerebral tissue slice including 4096 NVU blocks globally coupled with a space-filling vascular tree**. A two-dimensional Gauss input function is used to activate the center of the tissue blocks with an increased luminal agonist flux (0.18–0.4 μ M s^−1^).

Our simulations concentrate on the resulting linkage between the vasculature and the NVU embedded in a tissue block. The results are divided into three sections where the simulations provide the system when there is only (i) neuronal activation only (varying spatially with the tissue block), (ii) agonist activation only, and (iii) where is both agonist and neuronal activation. Figure 5 shows a 64 × 64 tissue block slice containing 4096 individual blocks. The case shown simulates an Gaussian distribution of agonist centered in the center of the block and inducing oscillation in each of the tissue blocks out to a defined radius. Color maps of the blocks simulate Ca^2+^ whilst the color of the vascular segments simulate bloodflow. A video of the full time-dependent simulation can be found in the Supplementary Material.

#### 3.2.1. Neuronal activiation only

**Figure 6:**
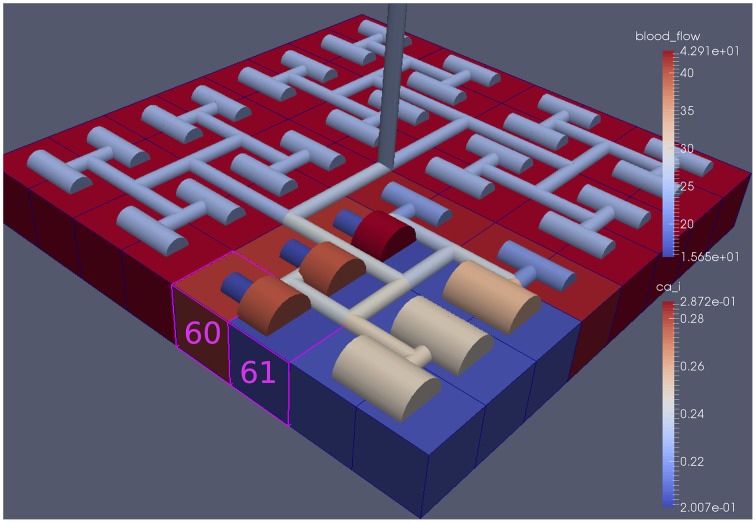
**A square of 9 tissue blocks (shown in blue) is activated with neuronal K^+^ release (***F***_***input***_ = 2.5)**.

Figure 6 shows the tissue block of size 8 × 8 where the neuron is activated in the area denoted by the 3 × 3 sub-block whose border is defined by cells numbered 60 (outside) and 61 (inside). The neuronal activation starts at *t* = 100 s. The color map on the vessel segments denotes blood flow whilst the color mapped onto the tissue blocks denotes cytosolic SMC Ca^2+^. For this particular part of the time dependent behavior the radius inside the activate area shows a constant and dilated set of arterial segments. As a response, vessels in the activated area dilate and due to flow conservation adjacent vessels outside get constricted. If both leaf vessels lay in the activated area their dilation is smaller than if in different areas. NVU tissue blocks and vasculature are bidirectionally coupled. The feedback from the radius to the NVU via the stretch-activated channels leads to a (small) change in Ca^2+^ also for the tissue blocks in the non-activated area.

**Figure 7:**
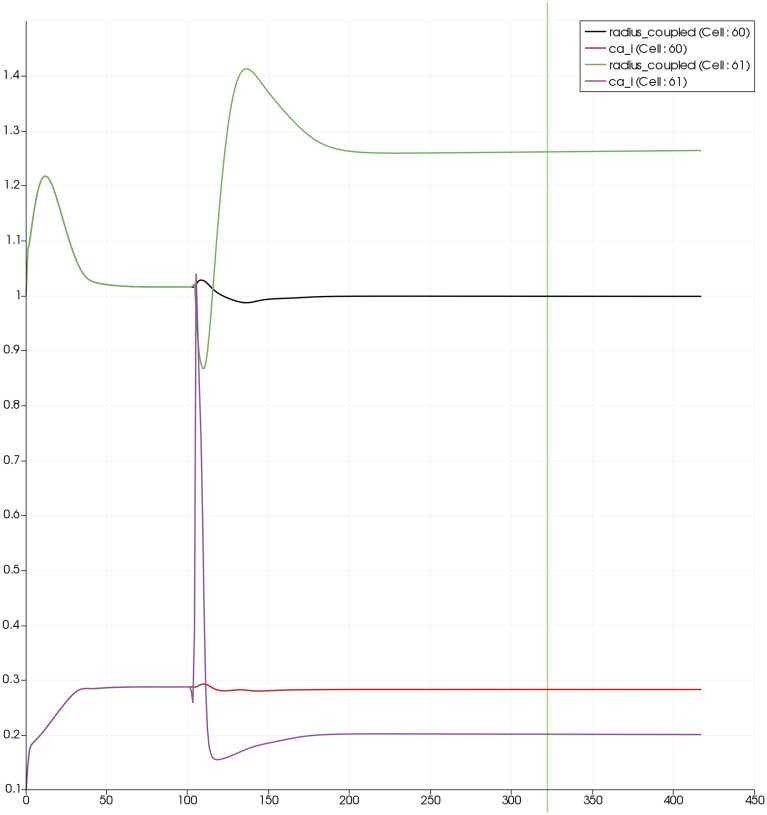
**SMC Ca^2+^ concentration changes in two adjacent tissue blocks and radius dynamics in the corresponding leaf branches**. Tissue block and leaf vessel 60 lay in a non-activated area and 61 in an area which is activated with a time-dependent K^+^ input (*F*_*input*_ = 2.5). NVU tissue blocks and vasculature are bidirectionally coupled (stretch-activated channels enabled).

Figure 7 shows the time dependent profiles for both Ca^2+^ and radius for the cells 60 and 61. At the start of the activation the radius of cell 61 (inside the activated area) shows a constriction and then a dilation to accommodate the increase in neuronal activity and hence oxygen consumption. The Ca^2+^ for cell 61 shows an initial increase in value but then decreases to a constant value. This is due to the action of the increase in K^+^ in the perivascular space and subsequent hyperpolarization of the SMC. The Ca^2+^ and radius in cell 60 (outside of the activated area) shows only a small perturbation with the radius reaching a constant value slightly lower than the steady state, due to the effect of the conservation of mass at the junction of the vessel segments that perfuse cells 60 and 61.

#### 3.2.2. Lumen agonist activiation only

**Figure 8:**
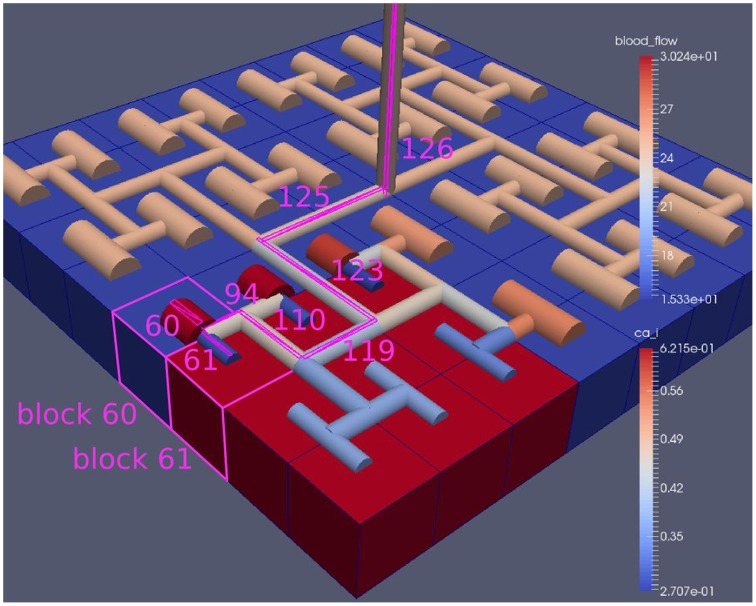
**A square of 9 tissue blocks is activated with an increased luminal agonist flux (0.4 μ M s^−1^)**. As a response, vessel radii in the activated area become oscillatory. The vessel segments are number from the root (126) to the leaf (60, 61) Radial diameters are shown proportional to their size with color mapped as proportional to blood flow. Tissue blocks are colored proportional to SMC Ca^2+^. NVU tissue blocks and vasculature are uncoupled (stretch-activated channels are disabled).

**Figure 9:**
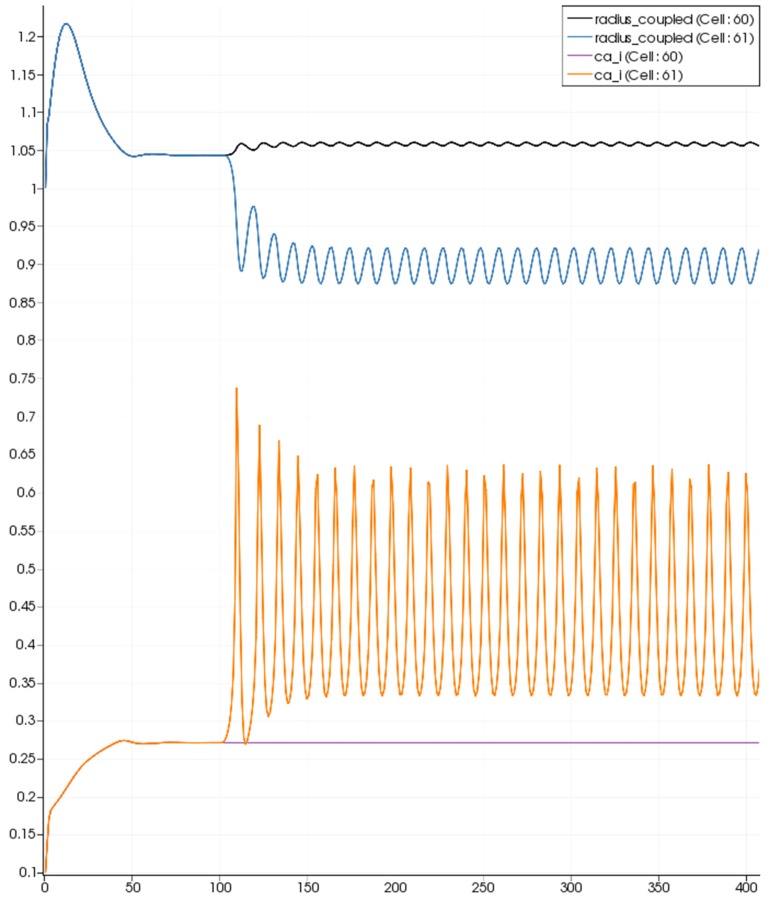
**SMC Ca^2+^ concentration changes in two adjacent tissue blocks and radius dynamics in the corresponding leaf branches**. Tissue block and leaf vessel 60 lay in a non-activated area and 61 in an area which is activated with a time-dependent luminal agonist flux (0.4 μ M s^−1^). NVU tissue blocks and vasculature are uncoupled (stretch-activated channels disabled).

**Figure 10:**
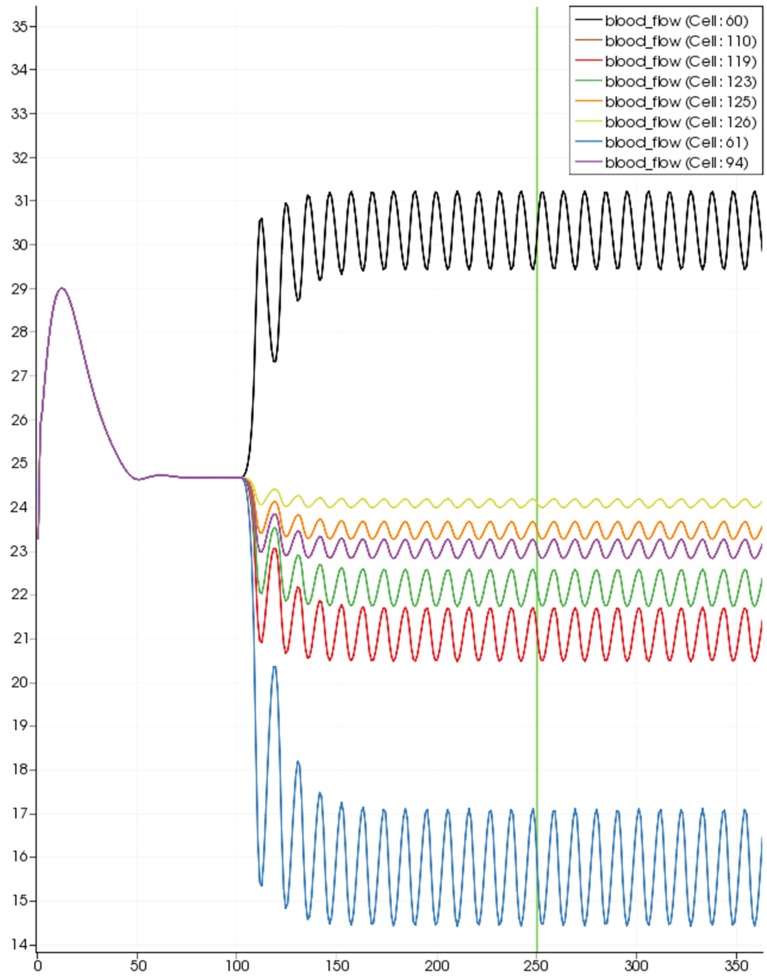
**SMC Ca^2+^ concentration time-dependent profiles in vessel segments (60, 61, 94, 110, 119, 123, 115, 126)**. Tissue block and leaf vessel 60 lay in a non-activated area and 61 in an area which is activated with a time-dependent luminal agonist flux (0.4 μ M s^−1^).

**Figure 11:**
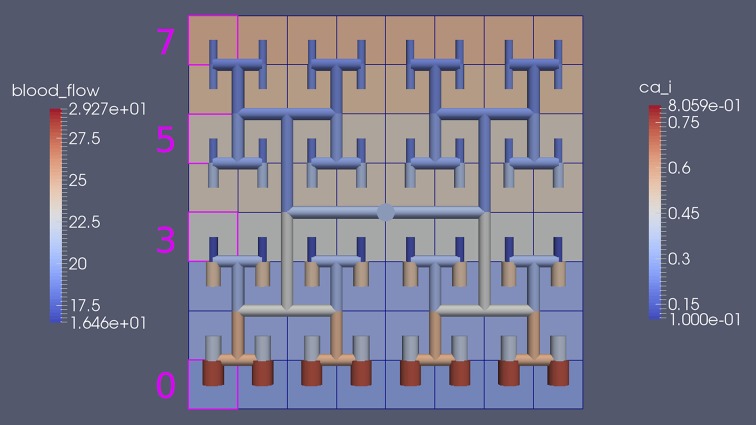
**Sixty-four tissue block with agonist profile is varying linearly from top to bottom of the tissue block**. The tissue color map simulates cytosolic SMC Ca^2+^ and the vascular color indicates bloodflow. The size of the radial segments indicate the actual radius of the vessel segment. Four blocks are highlighted and number 0, 3, 5, 7.

**Figure 12:**
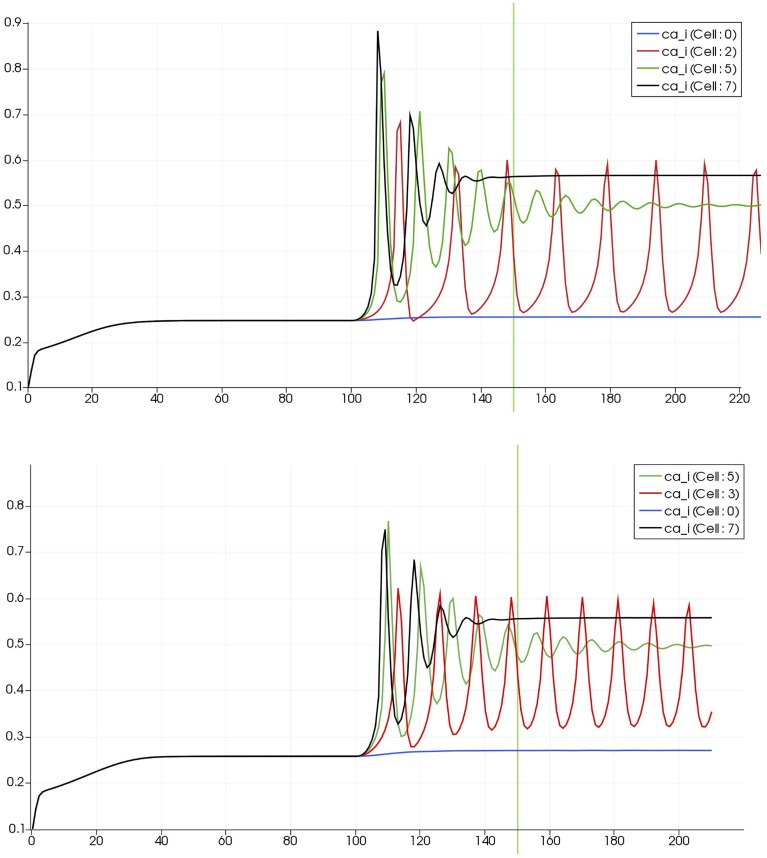
**Time dependent Ca^2+^ profiles in the four tissue blocks for the decoupled (top) and coupled (bottom) case shown as an overview in Figure 1**.

Figure 8 shows a detail of the same size 64 block (as shown in Figure 6) simulation where the vasculature has been decoupled from the NVU model. The small 9 × 9 block (red) has a constant agonist flux of 0.4 μ M s^−1^ whilst the remaining blocks (blue) have a constant value of 0.18 μ M s^−1^. Two blocks are also highlighted where one block lies within the high agonist domain (cell 61) and the other (cell 60) lies adjacent but inside the low agonist domain. Figure 9 shows the time-dependent profiles of radii in the two adjacent blocks (cells 60 and 61). Since the agonist flux of 0.4 μ M s^−1^ is high enough to include oscillation in the SMC, this is shown clearly in Figure 9 both in the radius of cell 61 (in the high flux domain) and in the cytosolic Ca^2+^ of the associated SMC. Additionally that although Ca^2+^ in the block outside of the high flux domain is constant the corresponding radius does exhibit small oscillations and indicates that the vasculature provides a form of coupling to other perfusing segments of the tree. Figure 8 shows numbered segments of the vasculature from the root segment (126) to the activated leaf of the H-tree (61). Figure 10 shows the corresponding bloodflow time dependent profiles for the number segments (60,…, 126). We see that the root (segment 126) has the lowest flow but contains the sum of all the lower mass flow rates but has the largest radius. Interestingly the blood flow to the two cells (60 and 61) both exhibit oscillations. Cell 61 has the lowest bloodflow and this is due to the fact that its Ca^2+^ has a high temporal average and thus induces a constriction. Conversely the cell adjacent but outside of the high agonist domain has the highest bloodflow in order to maintain mass conservation. The remaining segments exhibit oscillations due to pressure variations within the vasculature. Hence even though the vasculature and tissue blocks are effectively decoupled by forcing the pressure term in Equation (1.1) to be constant the tissue blocks outside of the activated domain are dynamic. Figure 11 shows a 64 tissue block with associated vasculature where the tissue color map simulates cytosolic SMC Ca^2+^ and the vascular color indicates bloodflow. The size of the radial segments indicate the actual radius of the vessel segment. In this case the agonist profile is varying linearly from top to bottom of the tissue block. Four blocks are highlighted and number 0, 3, 5, 7. Figure 12 (top) shows the time dependent Ca^2+^ profiles in the four tissue blocks for the decoupled case shown as an overview in Figure 1. The agonist profile is such that the 2 top block rows and that of the bottom 3 are such as to produce a steady steady state in the SMC Ca^2+^. Whilst all other rows induce oscillations in the SMC compartment of the NVU. Whilst Figure 12 (bottom) shows the same situation but where the coupling between NVU and vasculature is active, (i.e., the stretch channel has the radius and pressure terms changing). Comparison of the profiles show only a small change, most notably in the Ca^2+^ of cell block 3. The Ca^2+^ in the coupled case has a higher frequency and the profile is more sinusoidal in shape indicating that the calcium mediated channels are opening more quickly.

#### 3.2.3. Neuronal and agonist activation

**Figure 13:**
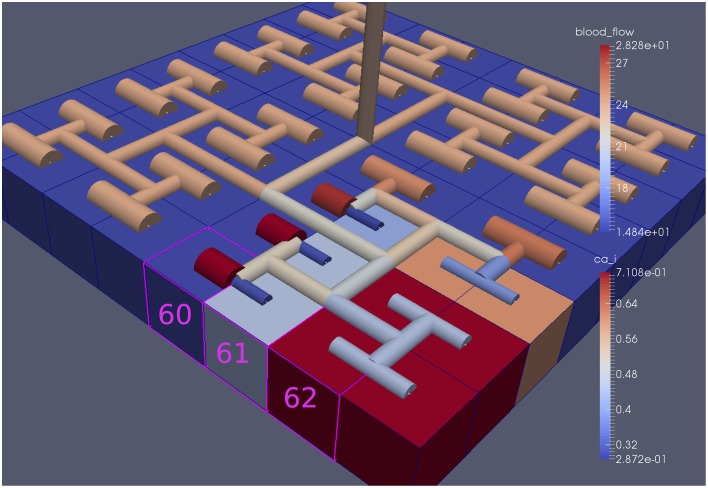
**Sixty-four block with both neuronal and agonist activation**. Highlighted blocks indicate outside (60), boundary (61), and inside (62) the activated domain.

**Figure 14:**
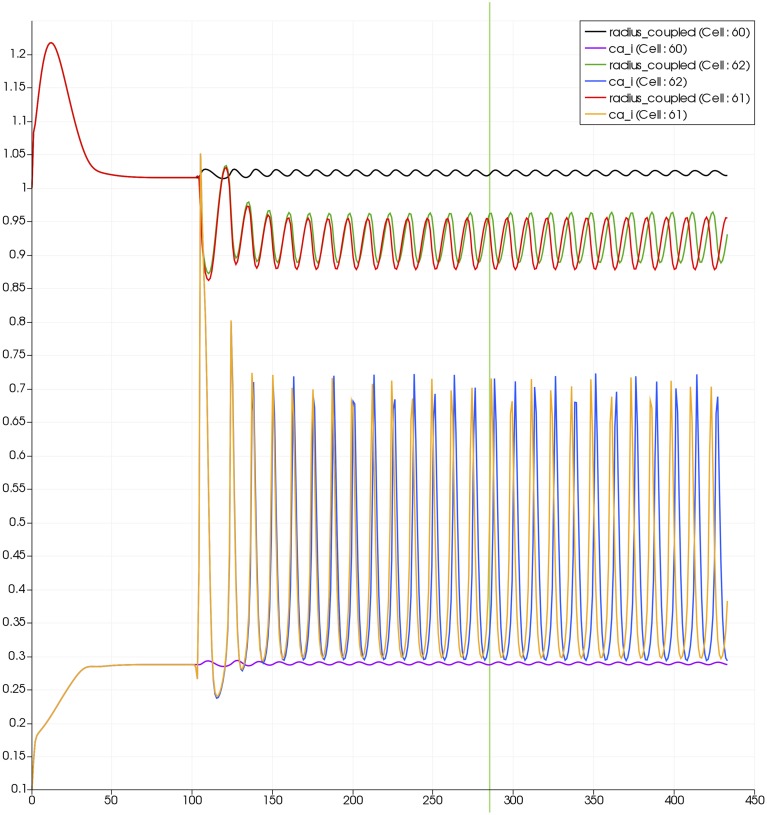
**Time dependent profiles for both radius and Ca^2+^ for cells numbered 60, 61, and 62**.

For this case both the neuron is activated by an increase in K^+^ in the synaptic cleft and an increase in agonist concentration over the highlighted area. Figure 13 shows the tissue blocks and numbered cells on the border of the activated area. Figure 14 shows for cells numbered 60, 61, and 62 the time dependent profiles for both radius and Ca^2+^. Tissue cell 60 (outside of the activated area) has the largest radius and the lowest Ca^2+^ perturbed only a small value from equilibrium but showing small amplitude oscillations. This is a particular effect of the vasculature on the system since if the vasculature was decoupled then the radius and Ca^2+^ would be of a constant value. For the cells (61 and 62) inside the activated area the time dependent profiles indicate that there exist a phase difference between both radius and Ca^2+^. The phase difference increases as time increases. Because the time averaged value of Ca^2+^ for both cells 61 and 62 is high the radius attains a constricted value compared to the equilibrium state.

## 4. Discussion

We have developed a coupled model which links a large dynamic vascular tree to a set of NVC units whose details can be found in Dormanns et al. (2015). Coupling is enabled by the inclusion of a variable pressure in the stretch-activated ion channel (see Equation 1.1). We have implemented three basic cases where a specific area of the tissue blocks is activated by either neuronal activation and the corresponding increase in perivascular K^+^ or the increase in agonist concentration or both. Scaling results show that strong scaling provides a near ideal profile. Weak scaling results indicate that wall clock time increases above 10% when the processor number increases from 8 to 256. This is not a large increase and again indicates that the parallel algorithm is close to optimal parallelization. Figure 4 shows that the algorithm provides a smaller wall clock time compared to the ideal as the number of state variables increases.

Figures 6–14 show a series of results from the three basic cases. They show that on the basis of the single stretch-activated channel per NVU block the effect of the vasculature coupling with the NVU shows only a weak association. However, there are interesting phenomena occurring on the boundaries of subdomains activated by either neuronal activity or agonist concentration. As expected for a neuronal activation only the solution reaches a steady state with segments outside of the activated area reaching values slightly smaller than the equilibrium. For the case with agonist induced only the Ca^2+^ in cells inside the activated area becomes oscillatory however, the radii of cells both inside and outside the activated area oscillate (albeit small for those outside). This shows the effect of the coupling through the pressure variations evaluated in the vascular tree although these are small. It is interesting to investigate the difference between profiles exhibited with and without coupling. Again only small differences occur and a slightly higher time averaged Ca^2+^ occurs with the coupled system. This is due to the increase in the open probability of the stretch mediated ion channel and inducing a larger flux of Ca^2+^ into the cytosol of the SMC and EC. In addition the oscillation profile differs between coupled and decoupled states with the time required to refill the cytosol with decreasing Ca^2+^ and increasing frequency with coupling.

### 4.1. Further work

We note that further work would benefit investigation in the following areas.

- At present the variation in arteriolar arteries only occurs at the leaves of the vascular tree. The future model will include variations in upstream segments of the vasculature utilizing a simple pressure balance as well as a myogenic model
- Investigation of oxygen flux from the perfusing vessel into the NVU providing energy to the ATPase pump of the neuron.
- The modeling of the transport of ions across tissue boundaries so as to simulate diffusion within the extracellular space.
- Investigation into the sensitivity in the parameters of the stretch activated channel.
- A further development of the NVU model to simulate the wall shear stress activated nitric oxide production and its subsequent effects on the cellular chemical pathways.
- Investigation of the wall shear stress induced ATP production and its influence on IP_3_ and subsequent pathways
- The inclusion of mechano-activated TRPV4 channels located on the endfeet membrane of the astrocyte.

## Author contributions

KD developed the NVU with the help of TD and RB. RB developed the parallel implementation. RB, KD, and TD analyzed the data. TD initially drafted the paper, editing and final approval was done by all authors.

## Funding

KD acknowledges the support of a UC Doctoral Scholarship from the University of Canterbury.

### Conflict of interest statement

The authors declare that the research was conducted in the absence of any commercial or financial relationships that could be construed as a potential conflict of interest.

## Supplementary material

A list of all equations and parameters of the NVU model.
