# Supplementary material for: Neurovascular coupling: a parallel implementation
Source: Front Comput Neurosci. 2015 Sep 15;9:109. doi: 10.3389/fncom.2015.00109 (PMC4569750; doi:10.3389/fncom.2015.00109)
Supplement: Supplementary file 1 [file DataSheet1.PDF]

Supplementary Material: Neurovascular Coupling: A Parallel Implementation

Katharina Dormanns [1,*], Richard Brown [2] and Tim David [1]

[1] *UC HPC Supercomputing Centre, University of Canterbury, Christchurch, New Zealand*
[2] *Institution of Mathematical Sciences, Massey University, Palmerston North, New Zealand*

Correspondence*:
Tim David
UC HPC Supercomputing Centre, University of Canterbury, Christchurch, New Zealand, tim.david@canterbury.ac.nz

1 EQUATIONS

1.1 THE NEURON AND ASTROCYTE MODEL

1.1.1 Input signals

Neuronal K^+ input signal (-):
For $t < t_0$ and $t > t_3$:

$$f(t) = 0 \tag{1.1}$$

For $t_0 \leq t \leq t_1$:

$$f(t) = F_{input} \frac{(\alpha+\beta-1)!}{(\alpha-1)!(\beta-1)!} \left(\frac{1-(t-t_0)}{\Delta t}\right)^{\beta-1} \left(\frac{t-t_0}{\Delta t}\right)^{\alpha-1} \tag{1.2}$$

For $t_1 < t \leq t_2$:

$$f(t) = 0 \tag{1.3}$$

For $t_2 < t \leq t_3$:

$$f(t) = -F_{input} \tag{1.4}$$

Parameter	Description	Value and unit	Reference
t_0	Start of neuronal pulse	200 s	
t_1	End of neuronal pulse	210 s	
t_2	Start of back-buffering	230 s	
t_3	End of back-buffering	240 s	
F_{input}	Amplitude scaling factor	2.5	
α	Gamma distribution constant	2	ME[1]
β	Gamma distribution constant	5	ME
Δt	Time-scaling factor	10 s	

1.1.2 Scaling

AC volume-area ratio (in m):

$$\frac{\mathrm{d}R_k}{\mathrm{d}t} = L_p(Na_k + K_k + Cl_k + HCO_{3_k} - Na_s - K_s - Cl_s - HCO_{3_s} + \frac{X_k}{R_k}) \tag{1.5}$$

SC volume-surface ratio (in m):

$$R_s = R_{tot} - R_k \tag{1.6}$$

Parameter	Description	Value and unit	Reference
L_p	Total water permeability per unit area of the astrocyte	2.1×10^{-9} m µM^{-1}s^{-1}	Østby et al. (2009)
X_k	Number of negatively charged impermeable ions trapped within the astrocyte divided by the astrocyte membrane area	12.41×10^{-3} µM m	Østby et al. (2009)
R_{tot}	Total volume surface ratio AC+SC	8.79×10^{-8} m	Østby et al. (2009)

1.1.3 Conservation Equations

Synaptic Cleft

K^+ concentration in the SC (times the SC volume-area ratio R_s; in µM m):

$$\frac{\mathrm{d}N_{K_s}}{\mathrm{d}t} = k_C f(t) - \frac{\mathrm{d}N_{K_k}}{\mathrm{d}t} \tag{1.7}$$

Na^+ concentration in the SC (times the SC volume-area ratio R_s; in µM m):

$$\frac{\mathrm{d}N_{Na_s}}{\mathrm{d}t} = -k_C f(t) - \frac{\mathrm{d}N_{Na_k}}{\mathrm{d}t} \tag{1.8}$$

HCO_3 concentration in the SC (times the SC volume-area ratio R_s; in µM m):

$$\frac{\mathrm{d}N_{HCO_{3_s}}}{\mathrm{d}t} = -\frac{\mathrm{d}N_{HCO_{3_k}}}{\mathrm{d}t} \tag{1.9}$$

Parameter	Description	Value and unit	Reference
k_C	Input scaling parameter	7.35×10^{-5} µM m s^{-1}	Østby et al. (2009)

[1] Model Estimation

Astrocyte
K^+ concentration in the AC (times the AC volume-area ratio R_k; in µM m):

$$\frac{\mathrm{d}N_{K_k}}{\mathrm{d}t} = -J_{K_k} + 2J_{NaK_k} + J_{NKCC1_k} + J_{KCC1_k} - J_{BK_k} \tag{1.10}$$

Na^+ concentration in the AC (times the AC volume-area ratio R_k; in µM m):

$$\frac{\mathrm{d}N_{Na_k}}{\mathrm{d}t} = -J_{Na_k} - 3J_{NaK_k} + J_{NKCC1_k} + J_{NBC_k} \tag{1.11}$$

HCO_3 concentration in the AC (times the AC volume-area ratio R_k; in µM m):

$$\frac{\mathrm{d}N_{HCO_{3_k}}}{\mathrm{d}t} = 2J_{NBC_k} \tag{1.12}$$

Cl concentration in the AC (times the AC volume-area ratio R_k; in µM m):

$$\frac{\mathrm{d}N_{Cl_k}}{\mathrm{d}t} = \frac{\mathrm{d}N_{Na_k}}{\mathrm{d}t} + \frac{\mathrm{d}N_{K_k}}{\mathrm{d}t} - \frac{\mathrm{d}N_{HCO_{3_k}}}{\mathrm{d}t} \tag{1.13}$$

Open probability of the BK channel (s^{-1}):

$$\frac{\mathrm{d}w_k}{\mathrm{d}t} = \phi_w \left(w_\infty - w_k\right) \tag{1.14}$$

Perivascular Space
K^+ concentration in the PVS (in µM):

$$\frac{\mathrm{d}K_p}{\mathrm{d}t} = \frac{J_{BK_k}}{R_k R_{pa}} + \frac{J_{KIR_i}}{R_{ps}} - R_{decay}(K_p - K_{p,min}) \tag{1.15}$$

Parameter	Description	Value and unit	Reference
R_{pa}	Volume ratio of PVS to AC	10^{-3} [-]	Nagelhus et al. (1999)
R_{ps}	Volume ratio of PVS to SMC	10^{-3} [-]	Nagelhus et al. (1999)
R_{decay}	Decay rate	10.05 s^{-1}	ME
$K_{p,min}$	Min K^+ concentration	3×10^3 µM	ME

1.1.4 Fluxes
K^+ flux (times the AC volume-area ratio R_k; in µM m s^{-1}):

$$J_{K_k} = \frac{g_{K_k}}{F}(v_k - E_{K_k}) \tag{1.16}$$

Na^+ flux (times the AC volume-area ratio R_k; in µM m s^{-1}):

$$J_{Na_k} = \frac{g_{Na_k}}{F}(v_k - E_{Na_k}) \tag{1.17}$$

Na^+ and HCO_3 flux through the NBC channel (times the AC volume-area ratio R_k; in µM m s^{-1}):

$$J_{NBC_k} = \frac{g_{NBC_k}}{F}\left(v_k - E_{NBC_k}\right) \tag{1.18}$$

Cl and K^+ flux through the KCC1 channel (times the AC volume-area ratio R_k; in µM m s^{-1}):

$$J_{KCC1_k} = C_{input}\frac{g_{KCC1_k}}{F}\frac{R_gT}{F}ln\left(\frac{K_sCl_s}{K_kCl_k}\right) \tag{1.19}$$

Na^+, K^+ and Cl flux through the NKCC1 channel (times the AC volume-area ratio R_k; in µM m s^{-1}):

$$J_{NKCC1_k} = C_{input}\frac{g_{NKCC1_k}}{F}\frac{R_gT}{F}ln\left(\frac{Na_sK_s{Cl_s}^2}{Na_kK_k{Cl_k}^2}\right) \tag{1.20}$$

Flux through the sodium potassium pump (times the AC volume-area ratio R_k; in µM m s^{-1}):

$$J_{NaK_k} = J_{NaK_{max}}\frac{{Na_k}^{1.5}}{{Na_k}^{1.5} + {K_{Na_k}}^{1.5}}\frac{K_s}{K_s + K_{K_s}} \tag{1.21}$$

K^+ flux through the BK channel (times the AC volume-area ratio R_k; in µM m s^{-1}):

$$J_{BK_k} = \frac{g_{BK_k}}{F}w_k\left(v_k - E_{BK_k}\right) \tag{1.22}$$

Parameter	Description	Value and unit	Reference
F	Faraday's constant	9.649×10^4 C mol^{-1}	
R_g	Gas constant	8.315 J $mol^{-1}K^{-1}$	
T	Temperature	300 K	
g_{K_k}	Specific ion conductance of potassium	40×10^3 $\Omega^{-1}m^{-2}$	Østby et al. (2009)
g_{Na_k}	Specific ion conductance of sodium	1.314×10^3 $\Omega^{-1}m^{-2}$	Østby et al. (2009)
g_{NBC_k}	Specific ion conductance of the NBC cotransporter	7.57×10^2 $\Omega^{-1}m^{-2}$	Østby et al. (2009)
g_{KCC1_k}	Specific ion conductance of the KCC1 cotransporter	10 $\Omega^{-1}m^{-2}$	Østby et al. (2009)
g_{NKCC1_k}	Specific ion conductance of the NKCC1 cotransporter	55.4 $\Omega^{-1}m^{-2}$	Østby et al. (2009)
$J_{NaK_{max}}$	Maximum flux through the NaKATPase pump	1.42×10^{-3} µM ms^{-1}	Østby et al. (2009)
g_{BK_k}	Specific ion conductance of the BK channel	1.16×10^3 $\Omega^{-1}m^{-2}$	Gonzalez-Fernandez (1994)
C_{input}	Block function to switch the channel on and off	0 ; 1 [-]	
K_{Na_k}	Michaelis-Menten constant	10^4 µM	
K_{K_s}	Michaelis-Menten constant	1.5×10^3 µM	

1.1.5 Additional Equations

Synaptic Cleft

Cl concentration (times the SC volume-area ratio R_s; in µM m):

$$N_{Cl_s} = N_{Na_s} + N_{K_s} - N_{HCO_{3s}} \tag{1.23}$$

Astrocyte
Membrane voltage of the AC (mV):

$$v_k = \frac{g_{Na_k}E_{Na_k} + g_{K_k}E_{K_k} + g_{Cl_k}E_{Cl_k} + g_{NBC_k}E_{NBC_k} + g_{BK_k}w_kE_{BK_k} - J_{NaK_k}F \times 10^3}{g_{Na_k} + g_{K_k} + g_{Cl_k} + g_{NBC_k} + g_{BK_k}w_k} \tag{1.24}$$

Nernst potential for the potassium channel (in mV):

$$E_{K_k} = \frac{R_gT}{z_KF}ln\left(\frac{K_s}{K_k}\right) \tag{1.25}$$

Nernst potential for the sodium channel (in mV):

$$E_{Na_k} = \frac{R_gT}{z_{Na}F}ln\left(\frac{Na_s}{Na_k}\right) \tag{1.26}$$

Nernst potential for the chloride channel (in mV):

$$E_{Cl_k} = \frac{R_gT}{z_{Cl}F}ln\left(\frac{Cl_s}{Cl_k}\right) \tag{1.27}$$

Nernst potential for the NBC channel (in mV):

$$E_{NBC_k} = \frac{R_gT}{z_{NBC}F}ln\left(\frac{Na_sHCO_{3_s}{}^2}{Na_kHCO_{3_k}{}^2}\right) \tag{1.28}$$

Nernst potential for the BK channel (in mV):

$$E_{BK_k} = \frac{R_gT}{z_KF}ln\left(\frac{K_p}{K_k}\right) \tag{1.29}$$

Equilibrium state BK-channel (-):

$$w_\infty = 0.5\left(1 + \tanh\left(\frac{v_k + v_6}{v_4}\right)\right) \tag{1.30}$$

Time constant associated with the opening of BK channels (in s^{-1}):

$$\phi_w = \psi_w \cosh\left(\frac{v_k + v_6}{2v_4}\right) \tag{1.31}$$

Parameter	Description	Value and unit	Reference
g_{Cl_k}	Specific ion conductance of chloride	0.879 Ω^{-1}m^{-2}	Østby et al. (2009)
z_K	Valence of a potassium ion	1	
z_{Na}	Valence of a sodium ion	1	
z_{Cl}	Valence of a chloride ion	-1	
z_{NBC}	Effective valence of the NBC cotransporter complex	-1	
v_6	Voltage associated with the opening of half the population	22 mV	Gonzalez-Fernandez (1994)
v_4	A measure of the spread of the distribution of the open probability of the BK channel	14.5 mV	Gonzalez-Fernandez (1994)
ψ_w	A characteristic time for the open probability of the BK channel	2.664 s^{-1}	Gonzalez-Fernandez (1994)

1.2 THE SMOOTH MUSCLE CELL AND ENDOTHELIAL CELL MODEL

1.2.1 Conservation Equations

Smooth muscle cell
Cytosolic [Ca^{2+}] in the SMC (in µM):

$$\begin{aligned}\frac{\mathrm{d}[Ca^{2+}]_i}{\mathrm{d}t} = J_{IP_{3i}} - J_{SR_{uptake_i}} + J_{CICR_i} - J_{extrusion_i} + J_{SR_{leak_i}} \dots \\ -J_{VOCC_i} + J_{Na/Ca_i} + 0.1 J_{stretch_i} + J^{SMC-EC}_{Ca^{2+}-coupling_i}\end{aligned} \tag{1.32}$$

[Ca^{2+}] in the SR of the SMC (in µM):

$$\frac{\mathrm{d}[\widehat{Ca}^{2+}]_i}{\mathrm{d}t} = J_{SR_{uptake_i}} - J_{CICR_i} - J_{SR_{leak_i}} \tag{1.33}$$

Membrane potential of the SMC (in mV):

$$\begin{aligned}\frac{\mathrm{d}v_i}{\mathrm{d}t} = \gamma_i(-J_{Na/K_i} - J_{Cl_i} - 2J_{VOCC_i} - J_{Na/Ca_i} - J_{K_i} \dots \\ -J_{stretch_i} - J_{KIR_i}) + V^{SMC-EC}_{coupling_i}\end{aligned} \tag{1.34}$$

Open state probability of calcium-activated potassium channels (dim.less):

$$\frac{\mathrm{d}w_i}{\mathrm{d}t} = \lambda_i \left(K_{act_i} - w_i\right) \tag{1.35}$$

IP$_3$ concentration om the SMC (in µM):

$$\frac{\mathrm{d}[IP_3]_i}{\mathrm{d}t} = J^{SMC-EC}_{IP_3-coupling_i} - J_{degrad_i} \tag{1.36}$$

K$^+$ concentration in the SMC (in µM):

$$\frac{\mathrm{d}[K_i^+]}{\mathrm{d}t} = J_{Na/K_i} - J_{KIR_i} - J_{K_i} \tag{1.37}$$

Parameter	Description	Value and unit	Reference
γ_i	Change in membrane potential by a scaling factor	1970 mV μM^{-1}	Koenigsberger et al. (2006)
λ_i	Rate constant for opening	45.0 s^{-1}	Koenigsberger et al. (2006)

Endothelial cell
Cytosolic Ca^{2+} concentration in the EC (in μM):

$$\frac{\mathrm{d}[Ca^{2+}]_j}{\mathrm{d}t} = J_{IP_{3j}} - J_{ER_{uptake_j}} + J_{CICR_j} - J_{extrusion_j} \cdots \\ + J_{ER_{leak_j}} + J_{cation_j} + J_{0_j} + J_{stretch_j} - J^{SMC-EC}_{Ca^{2+}-coupling_j} \tag{1.38}$$

Ca^{2+} concentration in the ER in the EC (in μM):

$$\frac{\mathrm{d}[\widehat{Ca}^{2+}]_j}{\mathrm{d}t} = J_{SR_{uptake_j}} - J_{CICR_j} - J_{SR_{leak_j}} \tag{1.39}$$

Membrane potential of the EC (in mV):

$$\frac{\mathrm{d}v_j}{\mathrm{d}t} = -\frac{1}{C_{m_j}}(J_{K_j} + J_{R_j}) + V^{SMC-EC}_{coupling_j} \tag{1.40}$$

IP$_3$ concentration of the EC (in μM):

$$\frac{\mathrm{d}[IP_3]_j}{\mathrm{d}t} = J_{EC,IP_3} - J_{degrad_j} - J^{SMC-EC}_{IP_3-coupling_j} \tag{1.41}$$

Parameter	Description	Value and unit	Reference
C_{m_j}	Membrane capacitance	25.8 pF	Koenigsberger et al. (2006)
J_{EC,IP_3}	IP$_3$ production rate	0.18; 0.4 μM s^{-1}	Koenigsberger et al. (2006)

1.2.2 Fluxes

Smooth muscle cell
Release of calcium from IP$_3$ sensitive stores in the SMC (in μM s^{-1}):

$$J_{IP_{3i}} = F_i \frac{[IP_3]_i^2}{K_{ri}^2 + [IP_3]_i^2} \tag{1.42}$$

Uptake of calcium into the sarcoplasmic reticulum (in μM s^{-1}):

$$J_{SR_{uptake_i}} = B_i \frac{[Ca^{2+}]_i^2}{c_{bi}^2 + [Ca^{2+}]_i^2} \tag{1.43}$$

Parameter	Description	Value and unit	Reference
F_i	Maximal rate of activation-dependent calcium influx	0.23 µM s^{-1}	Koenigsberger et al. (2006)
K_{ri}	Half-saturation constant for agonist-dependent calcium entry	1 µM	Koenigsberger et al. (2006)
B_i	SR uptake rate constant	2.025 µM s^{-1}	Koenigsberger et al. (2006)
c_{bi}	Half-point of the SR ATPase activation sigmoidal	1.0 µM	Koenigsberger et al. (2006)

Calcium-induced calcium release (CICR; in µM s^{-1}):

$$J_{CICR_i} = C_i \frac{[\widehat{Ca}^{2+}]_i^2}{s_{ci}^2 + [\widehat{Ca}^{2+}]_i^2} \frac{[Ca^{2+}]_i^4}{c_{ci}^4 + [Ca^{2+}]_i^4} \tag{1.44}$$

Calcium extrusion by Ca^{2+}-ATPase pumps (in µM s^{-1}):

Parameter	Description	Value and unit	Reference
C_i	CICR rate constant	55 µM s^{-1}	Koenigsberger et al. (2006)
s_{ci}	Half-point of the CICR Ca^{2+} efflux sigmoidal	2.0 µM	Koenigsberger et al. (2006)
c_{ci}	Half-point of the CICR activation sigmoidal	0.9 µM	Koenigsberger et al. (2006)

$$J_{extrusion_i} = D_i [Ca^{2+}]_i \left(1 + \frac{v_i - v_d}{R_{di}}\right) \tag{1.45}$$

Parameter	Description	Value and unit	Reference
D_i	Rate constant for Ca^{2+} extrusion by the ATPase pump	0.24 s^{-1}	Koenigsberger et al. (2006)
v_d	Intercept of voltage dependence of extrusion ATPase	-100.0 mV	Koenigsberger et al. (2006)
R_{di}	Slope of voltage dependence of extrusion ATPase.	250.0 mV	Koenigsberger et al. (2006)

Leak current from the SR (in µM s^{-1}):

$$J_{SR_{leak_i}} = L_i [\widehat{Ca}^{2+}]_i \tag{1.46}$$

Parameter	Description	Value and unit	Reference
L_i	Leak from SR rate constant	0.025 s^{-1}	Koenigsberger et al. (2006)

Calcium influx through VOCCs (in µM s^{-1}):

$$J_{VOCC_i} = G_{Cai} \frac{v_i - v_{Ca_{1i}}}{1 + exp(-\left[(v_i - v_{Ca_{2i}})/R_{Cai}\right])} \tag{1.47}$$

Flux of calcium exchanging with sodium in the Na^+Ca^{2+} exchange (in µM s^{-1}):

Parameter	Description	Value and unit	Reference
G_{Cai}	Whole-cell conductance for VOCCs	1.29×10^{-3} µM $mV^{-1}s^{-1}$	Koenigsberger et al. (2006)
$v_{Ca_{1i}}$	Reversal potential for VOCCs	100.0 mV	Koenigsberger et al. (2006)
$v_{Ca_{2i}}$	Half-point of the VOCC activation sigmoidal	-24.0 mV	Koenigsberger et al. (2006)
R_{Cai}	Maximum slope of the VOCC activation sigmoidal	8.5 mV	Koenigsberger et al. (2006)

$$J_{Na/Ca_i} = G_{Na/Ca_i} \frac{[Ca^{2+}]_i}{[Ca^{2+}]_i + c_{Na/Cai}} \left(v_i - v_{Na/Ca_i}\right) \tag{1.48}$$

Parameter	Description	Value and unit	Reference
$G_{Na/Cai}$	Whole-cell conductance for Na^+/Ca^{2+} exchange	3.16×10^{-3} µM $mV^{-1}s^{-1}$	Koenigsberger et al. (2006)
$c_{Na/Cai}$	Half-point for activation of Na^+/Ca^{2+} exchange by Ca^{2+}	0.5 µM	Koenigsberger et al. (2006)
$v_{Na/Cai}$	Reversal potential for the Na^+/Ca^{2+} exchanger	-30.0 mV	Koenigsberger et al. (2006)

Calcium flux through the stretch-activated channels in the SMC (in µM s^{-1}):

$$J_{stretch_i} = \frac{G_{stretch}}{1 + exp\left(-\alpha_{stretch}\left(\frac{\Delta pR}{h} - \sigma_0\right)\right)} \left(v_i - E_{SAC}\right) \tag{1.49}$$

Parameter	Description	Value and unit	Reference
$G_{stretch}$	Whole cell conductance for SACs	6.1×10^{-3} µM $mV^{-1}s^{-1}$	Koenigsberger et al. (2006)
$\alpha_{stretch}$	Slope of stress dependence of the SAC activation sigmoidal	7.4×10^{-3} $mmHg^{-1}$	Koenigsberger et al. (2006)
Δp	Pressure difference	30 mmHg	ME
σ_0	Half-point of the SAC activation sigmoidal	500 mmHg	Koenigsberger et al. (2006)
E_{SAC}	Reversal potential for SACs	-18 mV	Koenigsberger et al. (2006)

Flux through the sodium potassium pump (in µM s^{-1}):

$$J_{NaK_i} = F_{NaK} \tag{1.50}$$

Parameter	Description	Value and unit	Reference
F_{NaK}	Rate of the potassium influx by the sodium potassium pump	4.32×10^{-2} µM s^{-1}	Koenigsberger et al. (2006)

Chloride flux through the chloride channel (in µM s^{-1}):

$$J_{Cl_i} = G_{Cli}\left(v_i - v_{Cli}\right) \tag{1.51}$$

Parameter	Description	Value and unit	Reference
G_{Cli}	Whole-cell conductance for Cl$^-$ current	1.34×10^{-3} µM mV^{-1}s^{-1}	Koenigsberger et al. (2006)
v_{Cli}	Reversal potential for Cl$^-$ channels.	-25.0 mV	Koenigsberger et al. (2006)

Potassium flux through potassium channel (in µM s^{-1}):

$$J_{K_i} = G_{Ki} w_i \left(v_i - vK_i\right) \tag{1.52}$$

Parameter	Description	Value and unit	Reference
G_{Ki}	Whole-cell conductance for K$^+$ efflux.	4.46×10^{-3} µM mV^{-1}s^{-1}	Koenigsberger et al. (2006)
vK_i	Nernst potential	-94 mV	Koenigsberger et al. (2006)

Flux through KIR channels in the SMC (in µM s^{-1}):

$$J_{KIR_i} = \frac{F_{KIR_i} g_{KIR_i}}{\gamma_i}\left(v_i - v_{KIR_i}\right) \tag{1.53}$$

Parameter	Description	Value and unit	Reference
F_{KIR_i}	Scaling factor of potassium efflux through the KIR channel	750 mV µM^{-1}	Gonzalez-Fernandez (1994)

IP_3 degradation (in µM s^{-1}):

$$J_{degrad_i} = k_{di} I_i \tag{1.54}$$

Parameter	Description	Value and unit	Reference
k_{di}	Rate constant of IP_3 degradation	0.1 s^{-1}	Koenigsberger et al. (2006)

Endothelial cell

Release of calcium from IP_3-sensitive stores in the EC (in µM s^{-1}):

$$J_{IP_{3j}} = F_j \frac{[IP_3]_j^2}{K_{rj}^2 + [IP_3]_j^2} \tag{1.55}$$

Parameter	Description	Value and unit	Reference
F_j	Maximal rate of activation-dependent calcium influx	0.23 µM s^{-1}	Koenigsberger et al. (2006)
K_{rj}	Half-saturation constant for agonist-dependent calcium entry	1 µM	Koenigsberger et al. (2006)

Uptake of calcium into the endoplasmic reticulum (in µM s^{-1}):

$$J_{ER_{uptake_j}} = B_j \frac{[Ca^{2+}]_j^2}{c_{bj}^2 + [Ca^{2+}]_j^2} \tag{1.56}$$

Parameter	Description	Value and unit	Reference
B_j	ER uptake rate constant	0.5 µM s^{-1}	Koenigsberger et al. (2006)
c_{bj}	Half-point of the SR ATPase activation sigmoidal	1.0 µM	Koenigsberger et al. (2006)

Calcium-induced calcium release (CICR; in µM s^{-1}):

$$J_{CICR_j} = C_j \frac{[\widehat{Ca}^{2+}]_j^2}{s_{cj}^2 + [\widehat{Ca}^{2+}]_j^2} \frac{[Ca^{2+}]_j^4}{c_{cj}^4 + [Ca^{2+}]_j^4} \tag{1.57}$$

Parameter	Description	Value and unit	Reference
C_j	CICR rate constant	5 µM s^{-1}	Koenigsberger et al. (2006)
s_{cj}	Half-point of the CICR Ca^{2+} efflux sigmoidal	2.0 µM	Koenigsberger et al. (2006)
c_{cj}	Half-point of the CICR activation sigmoidal	0.9 µM	Koenigsberger et al. (2006)

Calcium extrusion by Ca^{2+}-ATPase pumps (in µM s^{-1}):

$$J_{extrusion_j} = D_j [Ca^{2+}]_j \tag{1.58}$$

Parameter	Description	Value and unit	Reference
D_j	Rate constant for Ca^{2+} extrusion by the ATPase pump	0.24 s^{-1}	Koenigsberger et al. (2005)

Calcium flux through the stretch-activated channels in the EC (in µM s^{-1}):

$$J_{stretch_j} = \frac{G_{stretch}}{1 + e^{-\alpha_{stretch}(\sigma - \sigma_0)}} (v_j - E_{SAC}) = \frac{G_{stretch}}{1 + e^{-\alpha_{stretch}\left(\frac{\Delta pR}{h} - \sigma_0\right)}} (v_j - E_{SAC}) \tag{1.59}$$

Parameter	Description	Value and unit	Reference
$G_{stretch}$	The whole cell conductance for SACs	6.1×10^{-3} µM $mV^{-1}s^{-1}$	Koenigsberger et al. (2006)
$\alpha_{stretch}$	Slope of stress dependence of the SAC activation sigmoidal	7.4×10^{-3} $mmHg^{-1}$	Koenigsberger et al. (2006)
Δp	Pressure difference	30 mmHg	ME
σ_0	Half-point of the SAC activation sigmoidal	500 mmHg	Koenigsberger et al. (2006)
E_{SAC}	The reversal potential for SACs	-18 mV	Koenigsberger et al. (2006)

Leak current from the ER (in µM s^{-1}):

$$J_{ER_{leak_j}} = L_j[\widehat{Ca}^{2+}]_j \tag{1.60}$$

Parameter	Description	Value and unit	Reference
L_j	Rate constant for Ca^{2+} leak from the ER	0.025 s^{-1}	Koenigsberger et al. (2006)

Calcium influx through nonselective cation channels (in µM s^{-1}):

$$J_{cation_j} = G_{cat_j}(E_{Ca_j} - v_j)\frac{1}{2}\left(1 + \tanh\left(\frac{\log_{10}[Ca^{2+}]_j - m_{3_{cat_j}}}{m_{4_{cat_j}}}\right)\right) \tag{1.61}$$

Parameter	Description	Value and unit	Reference
G_{catj}	Whole-cell cation channel conductivity	6.6×10^{-4} µM $mV^{-1}s^{-1}$	Koenigsberger et al. (2006)
E_{Caj}	Ca^{2+} equilibrium potential	50 mV	Koenigsberger et al. (2006)
$m_{3_{catj}}$	Model constant	-0.18 µM	Koenigsberger et al. (2006)
$m_{4_{catj}}$	Model constant	0.37 µM	Koenigsberger et al. (2006)

Potassium efflux through the $J_{BK_{Caj}}$ channel and the $J_{SK_{Caj}}$ channel (in µM s^{-1}):

$$J_{K_j} = G_{totj}(v_j - v_{Kj}) \left(J_{BK_{Caj}} + J_{SK_{Caj}} \right) \tag{1.62}$$

Parameter	Description	Value and unit	Reference
G_{totj}	Total potassium channel conductivity.	6927 pS	Koenigsberger et al. (2006)
v_{Kj}	K^+ equilibrium potential	-80.0 mV	Koenigsberger et al. (2006)

Potassium efflux through the $J_{BK_{Caj}}$ channel (in µM s^{-1}):

$$J_{BK_{Caj}} = 0.2 \left(1 + \tanh \left(\frac{(\log_{10}[Ca^{2+}]_j - c)(v_j - b_j) - a_{1j}}{m_{3bj}(v_j + a_{2j}(\log_{10}[Ca^{2+}]_j - c) - b_j)^2 + m_{4bj}} \right) \right) \tag{1.63}$$

Potassium efflux through the $J_{SK_{Caj}}$ channel (in µM s^{-1}):

$$J_{SK_{Caj}} = 0.3 \left(1 + \tanh \left(\frac{\log_{10}[Ca^{2+}]_j - m_{3sj}}{m_{4sj}} \right) \right) \tag{1.64}$$

Parameter	Description	Value and unit	Reference
c	Model constant, further explanation see reference	-0.4 µM	Koenigsberger et al. (2006)
b_j	Model constant, further explanation see reference	-80.8 mV	Koenigsberger et al. (2006)
a_{1j}	Model constant, further explanation see reference	53.3 µM mV	Koenigsberger et al. (2006)
a_{2j}	Model constant, further explanation see reference	53.3 mV µM^{-1}	Koenigsberger et al. (2006)
m_{3bj}	Model constant, further explanation see reference	1.32×10^{-3} µM mV^{-1}	Koenigsberger et al. (2006)
m_{4bj}	Model constant, further explanation see reference	0.30 µM mV	Koenigsberger et al. (2006)
m_{3sj}	Model constant, further explanation see reference	-0.28 µM	Koenigsberger et al. (2006)
m_{4sj}	Model constant, further explanation see reference	0.389 µM	Koenigsberger et al. (2006)

Residual current regrouping chloride and sodium current flux (in µM s^{-1}):

$$J_{R_j} = G_{R_j}(v_j - v_{restj}) \tag{1.65}$$

Parameter	Description	Value and unit	Reference
G_{R_j}	Residual current conductivity	955 pS	Koenigsberger et al. (2006)
v_{restj}	Membrane resting potential	-31.1 mV	Koenigsberger et al. (2006)

IP_3 degradation (in µM s^{-1}):

$$J_{degrad_j} = k_{dj}[IP_3]_j \tag{1.66}$$

Parameter	Description	Value and unit	Reference
k_{dj}	Rate constant of IP_3 degradation	0.1 s^{-1}	Koenigsberger et al. (2006)

1.2.3 Coupling
Heterocellular electrical coupling between SMCs en ECs (in mV s^{-1}):

$$V^{SMC-EC}_{coupling_i} = -G_{coup}(v_i - v_j) \tag{1.67}$$

Heterocellular IP_3 coupling between SMCs and ECs (in µM s^{-1}):

$$J^{SMC-EC}_{IP_3-coupling_i} = -P_{IP_3}([IP_3]_i - [IP_3]_j) \tag{1.68}$$

Calcium coupling with EC (inµM s^{-1}):

$$J^{SMC-EC}_{Ca^{2+}-coupling_i} = -P_{Ca^{2+}}([Ca^{2+}]_i - [Ca^{2+}]_j) \tag{1.69}$$

Parameter	Description	Value and unit	Reference
G_{coup}	Heterocellular electrical coupling coefficient	0.5 s^{-1}	ME
P_{IP_3}	Heterocellular IP_3 coupling coefficient	0.05 s^{-1}	Koenigsberger et al. (2006)
$P_{Ca^{2+}}$	Heterocellular $P_{Ca^{2+}}$ coupling coefficient	0.05 s^{-1}	Koenigsberger et al. (2006)

1.2.4 Additional Equations Equilibrium distribution of open channel states for the voltage and calcium activated potassium channels (dimensionless):

$$K_{act_i} = \frac{\left([Ca^{2+}]_i + c_{wi}\right)^2}{\left([Ca^{2+}]_i + c_{wi}\right)^2 + \beta_i exp(-\left([v_i - v_{Ca_{3i}}\right]/R_{Ki}))} \tag{1.70}$$

Nernst potential of the KIR channel in the SMC (in mV):

$$v_{KIR_i} = z_1 K_p - z_2 \tag{1.71}$$

Conductance of KIR channel (in µM mV^{-1} s^{-1}):

$$g_{KIR_i} = exp(z_5 v_i + z_3 K_p - z_4) \tag{1.72}$$

Parameter	Description	Value and unit	Reference
c_{wi}	Translation factor for Ca^{2+} dependence of K_{Ca} channel activation sigmoidal.	0.0 µM	Koenigsberger et al. (2006)
β_i	Translation factor for membrane potential dependence of K_{Ca} channel activation sigmoidal.	0.13 μM^2	Koenigsberger et al. (2006)
$v_{Ca_{3i}}$	Half-point for the K_{Ca} channel activation sigmoidal.	-27 mV	Koenigsberger et al. (2006)
R_{Ki}	Maximum slope of the K_{Ca} activation sigmoidal.	12 mV	Koenigsberger et al. (2006)
z_1	Model estimation for membrane voltage KIR channel	4.5×10^3 mV μM^{-1}	(Filosa et al., 2006)
z_2	Model estimation for membrane voltage KIR channel	112 mV	(Filosa et al., 2006)
z_3	Model estimation for the KIR channel conductance	4.2×10^2 $mV^{-1}s^{-1}$	(Filosa et al., 2006)
z_4	Model estimation for the KIR channel conductance	12.6 µM $mV^{-1}s^{-1}$	(Filosa et al., 2006)
z_5	Model estimation for the KIR channel conductance	-7.4×10^{-2} µM $mV^{-2}s^{-1}$	(Filosa et al., 2006)

1.3 THE CONTRACTION MODEL

Fraction of free phosphorylated cross-bridges (dimensionless):

$$\frac{\mathrm{d}[Mp]}{\mathrm{d}t} = K_4[AMp] + K_1[M] - (K_2 + K_3)[Mp] \tag{1.73}$$

Fraction of attached phosphorylated cross-bridges (dimensionless):

$$\frac{\mathrm{d}[AMp]}{\mathrm{d}t} = K_3[Mp] + K_6[AM] - (K_4 + K_5)[AMp] \tag{1.74}$$

Fraction of attached dephosphorylated cross-bridges (dimensionless):

$$\frac{\mathrm{d}[AM]}{\mathrm{d}t} = K_5[AMp] - (K_7 + K_6)[AM] \tag{1.75}$$

Fraction of free non-phosphorylated cross-bridges (dimensionless):

$$[M] = 1 - [AM] - [AMp] - [Mp] \tag{1.76}$$

Rate constants that represent phosphorylation of M to Mp and of AM to AMp by the active myosin light chain kinase (MLCK), respectively (in s^{-1}):

$$K_1 = K_6 = \gamma_{cross}[Ca^{2+}]_i^{n_{cross}} \tag{1.77}$$

Parameter	Description	Value and unit	Reference
K_2	Rate constant for dephosphorylation (of Mp to M) by myosin light-chain phosphatase (MLCP)	0.5 s^{-1}	Hai and Murphy (1989)
K_3	Rate constants representing the attachment/detachment of fast cycling phosphorylated crossbridges	0.4 s^{-1}	Hai and Murphy (1989)
K_4	Rate constants representing the attachment/detachment of fast cycling phosphorylated crossbridges	0.1 s^{-1}	Hai and Murphy (1989)
K_5	Rate constant for dephosphorylation (of AMp to AM) by myosin light-chain phosphatase (MLCP)	0.5 s^{-1}	Hai and Murphy (1989)
K_7	Rate constant for latch-bridge detachment	0.1 s^{-1}	Hai and Murphy (1989)
γ_{cross}	Sensitivity of the contractile apparatus to calcium	17 μM^{-3} s^{-1}	Koenigsberger et al. (2005)
n_{cross}	Fraction constant of the phosphorylation crossbridge	3 $[-]$	Koenigsberger et al. (2005)

1.4 THE MECHANICAL MODEL

Wall thickness of the vessel (in µm):

$$h = 0.1R \tag{1.78}$$

Fraction of attached myosin cross-bridges (dimensionless):

$$F_r = [AM_p] + [AM] \tag{1.79}$$

Vessel radius (in µm):

$$\frac{\mathrm{d}R}{\mathrm{d}t} = \frac{R_{0_{pas}}}{\eta}\left(\frac{RP_T}{h} - E(F_r)\frac{R - R_0(F_r)}{R_0(F_r)}\right) \tag{1.80}$$

with:

$$E(F_r) = E_{pas} + F_r\left(E_{act} - E_{pas}\right) \tag{1.81}$$

$$R_0(F_r) = R_{0_{pas}} + F_r(\alpha - 1)R_{0_{pas}} \tag{1.82}$$

Parameter	Description	Value and unit	Reference
η	viscosity	10^4 Pa s	Koenigsberger et al. (2006)
$R_{0_{pas}}$	Radius of the vessel when passive and no stress is applied	20 µm	ME
P_T	Transmural pressure	4×10^3 Pa	ME
E_{pas}	Young's moduli for the passive vessel	66×10^3 Pa	Gore and Davis (1984)
E_{act}	Additional component of the Young's moduli when vessel is active	167×10^3 Pa	Gore and Davis (1984)
α	Scaling factor initial radius	0.6	Gore and Davis (1984)

REFERENCES

Østby I, Øyehaug L, Einevoll GT, Nagelhus EA, Plahte E, Zeuthen T, et al. Astrocytic Mechanisms Explaining Neural-Activity-Induced Shrinkage of Extraneuronal Space. *PLoS Computational Biology* **5** (2009) 1–12.

Nagelhus E, Horio Y, Inanobe A. Immunogold evidence suggests that coupling of K+ siphoning and water transport in rat retinal muller cells is mediated by a coenrichment of kir4. 1 and aqp4 in specific membrane domains. *Glia* **63** (1999) 47–54.

Gonzalez-Fernandez B JM Ermentrout. On the Origin and Dynamics of the Vasomotion of Small Arteries. *Mathematical Biosciences* **167** (1994) 127–167.

Koenigsberger M, Sauser R, Bény JL, Meister JJ. Effects of arterial wall stress on vasomotion. *Biophysical journal* **91** (2006) 1663–74. doi:10.1529/biophysj.106.083311.

Koenigsberger M, Sauser R, Bény JL, Meister JJ. Role of the endothelium on arterial vasomotion. *Biophysical journal* **88** (2005) 3845–54. doi:10.1529/biophysj.104.054965.

Filosa Ja, Bonev AD, Straub SV, Meredith AL, Wilkerson MK, Aldrich RW, et al. Local potassium signaling couples neuronal activity to vasodilation in the brain. *Nature neuroscience* **9** (2006) 1397–1403. doi:10.1038/nn1779.

Hai CM, Murphy RA. Ca^{2+}, Crossbridge Phosphorylation, and Contraction. *Annual Review of Physiology* **51** (1989) 285–298.

Gore RW, Davis MJ. Mechanics of Smooth Muscle in Isolated Single Microvessels. *Annals of Bioemdical Engineering* **12** (1984) 511–520.
